# A Hierarchical Short Microneedle-Cupping Dual-Amplified Patch Enables Accelerated, Uniform, Pain-Free Transdermal Delivery of Extracellular Vesicles

**DOI:** 10.1007/s40820-025-01853-7

**Published:** 2025-07-23

**Authors:** Minwoo Song, Minji Ha, Sol Shin, Minjin Kim, Soyoung Son, Jihyun Lee, Gui Won Hwang, Jeongyun Kim, Van Hieu Duong, Jae Hyung Park, Changhyun Pang

**Affiliations:** 1 School of Chemical Engineering, Sungkyunkwan University, 2066 Seobu-ro, Jangan-gu, Suwon, 16419 Republic of Korea; 2 Department of Health Sciences and Technology, SAIHST, Sungkyunkwan University, Seoul, 06355 Republic of Korea; 3 Biomedical Institute for Convergence at SKKU (BICS), Sungkyunkwan University, Suwon, 16419 Republic of Korea

**Keywords:** Biomimetics, Cupping, Microneedle, Transdermal patch, Extracellular vesicles

## Abstract

Microneedles (MNs) have been extensively investigated for transdermal delivery of large-sized drugs, including proteins, nucleic acids, and even extracellular vesicles (EVs). However, for their sufficient skin penetration, conventional MNs employ long needles (≥ 600 μm), leading to pain and skin irritation. Moreover, it is critical to stably apply MNs against complex skin surfaces for uniform nanoscale drug delivery. Herein, a dually amplified transdermal patch (MN@EV/SC) is developed as the stem cell-derived EV delivery platform by hierarchically integrating an octopus-inspired suction cup (SC) with short MNs (≤ 300 μm). While leveraging the suction effect to induce nanoscale deformation of the stratum corneum, MN@EV/SC minimizes skin damage and enhances the adhesion of MNs, allowing EV to penetrate deeper into the dermis. When MNs of various lengths are applied to mouse skin, the short MNs can elicit comparable corticosterone release to chemical adhesives, whereas long MNs induce a prompt stress response. MN@EV/SC can achieve a remarkable penetration depth (290 µm) for EV, compared to that of MN alone (111 µm). Consequently, MN@EV/SC facilitates the revitalization of fibroblasts and enhances collagen synthesis in middle-aged mice. Overall, MN@EV/SC exhibits the potential for skin regeneration by modulating the dermal microenvironment and ensuring patient comfort.

**Supplementary Information:**

The online version contains supplementary material available at 10.1007/s40820-025-01853-7.

## Highlights

- A bio-inspired dual-amplified patch (MN@EV/SC) was fabricated by integrating extracellular vesicle-loaded microneedles with a soft suction chamber for effective transdermal delivery.
- The MN@EV/SC system achieved an exceptional penetration depth of 290 μm, while minimizing plasma corticosterone levels with short microneedles, ensuring patient comfort through pain-free application.
- This system showed the potential for dermatological application by revitalizing dermal fibroblasts, and enhancing collagen synthesis through effective delivery of extracellular vesicles while preserving their biological functionality.

**Supplementary Information:**

The online version contains supplementary material available at 10.1007/s40820-025-01853-7.

## Graphical abstract

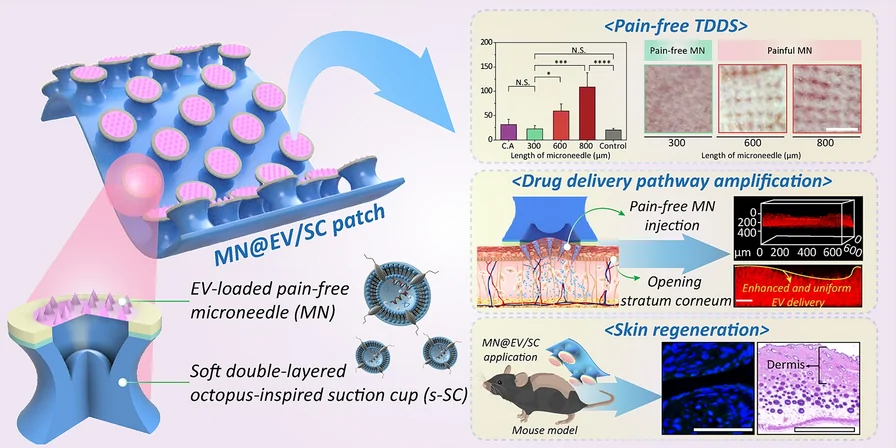

## Introduction

Transdermal drug delivery (TDD) has emerged as an efficacious alternative to other routes of administration since they facilitate drug delivery via bypassing the first-pass metabolism [1, 2]. In particular, this approach has garnered attention due to its benefits for patients in terms of convenience and compliance, while addressing the limitations associated with other routes such as discomfort, infection risk, poor drug absorption, and difficulty in dosage control [3]. However, the traditional TDD is often impeded by the structural elements of the skin, acting as a barrier to drug penetration, including the stratum corneum (ST), the epidermis, and the basement membrane [2, 4]. The ST permits the passage of only small molecules (typically under 500 Da) with sufficient affinity for the lipids present in the outer layer. Meanwhile, the hydrophilic nature of the epidermis precipitates hydrophobic drugs, and the basement membrane further precludes the subsequent transport of large molecules (> 10 nm in diameter), resulting in their retention within the epidermis [5]. While a variety of biologics (e.g., proteins, nucleic acids, and especially extracellular vesicles) have emerged as promising therapeutics for dermatological applications [6], they possess a larger size and more intricate structure which render them more vulnerable to the constraints imposed by the natural barrier of the skin in transdermal delivery systems [7, 8]. As they must access the deeper regions of the skin to exert their therapeutic effects, their topical administration has been highly challenging.

In recent years, much effort has been made to enhance drug penetration through the use of microneedles (MNs) or external stimuli, such as electric potential differences and mechanical pressure [9–13]. Particularly, the MNs enable sufficient penetration of a broad range of drugs by forming microchannels in the skin layer, irrespective of their molecular weights and hydrophilicity [14, 15]. Furthermore, the MNs can be combined with other topical administration technologies such as iontophoresis and ultrasound [16, 17]. Notwithstanding, there are challenges for clinical applications of the MNs, primarily ascribed to their dependency on the needle length: Typically, long MNs (≥ 600 μm) are used to ensure sufficient penetration, which often results in nagging discomfort and skin irritation [18, 19]. Moreover, the distinctive characteristics of the skin, including its non-flat surface and intricate topography (e.g., hierarchical wrinkles, glands, and hairs) [20], result in inconsistent and poor adhesion of MN patches [21, 22]. Therefore, maintaining uniform drug delivery around the patches and ensuring that the MNs remain intact during adhesion are critical issues.

In addition to MN-based systems, suction devices that employ hypobaric pressure to stretch the skin and in turn, enhance transdermal drug delivery by creating deformations in the skin, have demonstrated potential in improving therapeutic modalities such as photothermal therapy, gene expression, and vaccination [12, 23, 24]. However, the utilization of these technologies generally necessitates the employment of external devices, which consequently constrains accessibility and convenience for regular use [25]. Thus, there is an urgent need for a straightforward approach that does not rely on electronics or mechanical components. In order to address this challenge, we had developed bio-inspired architecture-based miniaturized suction cups (SCs) that mimic natural adhesion mechanisms [26, 27]. These SCs generate localized hypobaric pressure, enhancing skin permeability with only thumb pressure. In light of these considerations, integrating hypobaric pressure-induced deformation with short microneedles offers a promising solution.

**Fig. 1:**
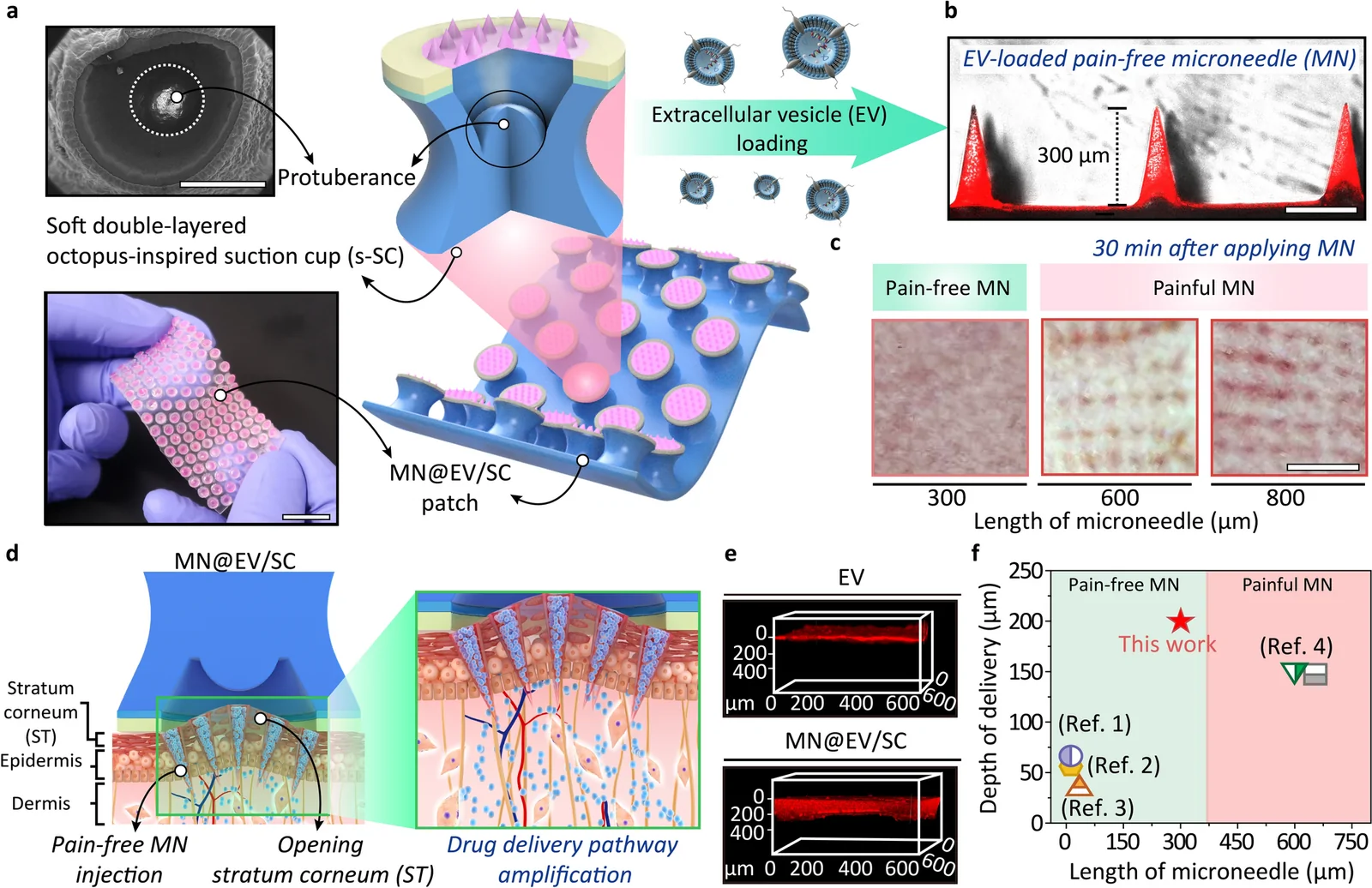
Dual-amplified transdermal patch system. **a** Photograph and schematic of MN@EV/SC for localized drug delivery. The inset shows SEM image of the forelegs of octopus vulgaris (left upper). Scale bar, 3 mm. Flexibility of MN@EV/SC is shown by typical images (left lower). Scale bar, 1 cm. Each suction cup is equipped with MNs (right). **b** 2D-reconstructed images of MN@EV. Scale bar, 300 μm. **c** Evaluation of skin disturbance by MNs with different lengths applied on mouse skin. Scale bar, 500 μm. **d** Mechanism of nanoscale deformations in skin layer and EV distribution behavior through dual amplification of MN@EV/SC. The inset shows the zoomed in details of the MN@EV/SC patched onto the skin. **e** Representative confocal images showing the distribution depth of EVs across skin layers. **f** Comparison of drug delivery depth and needle length between the MN@EV/SC developed in this work and MN-based drug delivery systems reported in the literature

Herein, we designed a bio-inspired, user-friendly, pain-free all-in-one system that hierarchically integrates short dissolving MNs with a cupping patch to achieve uniform drug delivery of EV to the target site with minimal discomfort and eliminating the necessity for bulky electronics or mechanical components. The dual-amplification patch (MN/SC), consisting of MN integrated into soft double-layered SC (s-SC), offers robust adhesion even on wet or uneven skin surfaces (Fig. 1a). EV-encapsulated MN (MN@EV) of 300 μm length at the skin interface minimize skin damage and the formation of micro-wounds. Simultaneously, the suction effect on the top of the patch device creates a temporary negative pressure on the skin, leading to nanoscale deformation of the stratum corneum and enhancing MN permeation and adhesion (Fig. 1b, c). This mechanical deformation increases uniform skin permeability, allowing EV to penetrate deeper dermal layers without the need for longer needles, invasive techniques, or electric components. The short MN was fabricated by encapsulating EV, which enabled sustained release of EV and preserved their functionality, allowing effective skin repair (Fig. 1d). To assess the potential of this system, comprehensive investigations were conducted using multiple models, including in vitro cell cultures, ex vivo skin tissues, and in vivo animal models. The EV-loaded MN/SC (MN@EV/SC) exhibited noteworthy efficiency and safety in EV delivery, enabling shorter MNs to attain delivery depths comparable to that of longer MNs while avoiding discomfort (Figs. 1e, f and S1). The proposed technique is a promising platform for macromolecular drug delivery for both anti-aging and therapeutic applications, providing a balance between efficacy and patient comfort.

## Experimental Section

### Materials

Trichloro(octadecyl)silane (ODTS) was purchased from Sigma-Aldrich (St. Louis, USA). Ecoflex 0030 was purchased from Smooth-On, Inc. (PA, USA). (Tridecafluoro-1,1,2,2-tetrahydrooctyl)-trichlorosilane (FOTCS) was obtained from Gelest (Morrisville, NY, USA). Microneedle master molds were purchased from MicroPoint Technologies (Singapore). The corticosterone (CORT) ELISA kit, MMP-2 ELISA kit, goat anti-rabbit secondary antibody, and anti-Col I antibody were purchased from Abcam (MA, USA). Tangential capsules were purchased from Pall Corporation (NY, USA). RPMI-1640 medium was purchased from Capricorn Scientific GmbH (Ebsdorfergrund, Germany). Dulbecco’s phosphate-buffered saline (DPBS), antibiotic–antimycotic (AA) solution, and trypsin–EDTA were obtained from Welgene (Gyeongsan, Korea). Transwell with 8.0 µm pore polycarbonate membrane insert was purchased from Corning (NY, USA). Sircol-soluble collagen and elastin assay kits were obtained from Biocolor (Carrickfergus, UK). The chemiluminescent substrates for western blotting were purchased from Thermo Fisher Scientific (Waltham, MA, USA). The FITC anti-mouse Ki-67 antibody and Flamma 675 NHS ester were obtained from Bioacts (Incheon, Korea). All other chemicals were commercially available and used without further purification.

### Preparation of MN@EV/SC

#### Fabrication of Octopus Inspired SC

3D printed master mold was prepared using a 3D computer-aided design software (Autodesk Fusion 360, Autodesk Inc.). The printed master mold was treated in a 1% diluted self-assembled monolayer (SAM) solution (trichloro(octadecyl)silane (ODTS); Sigma-Aldrich Inc.) of hexane as a solvent for 1 h at 25 °C and then baked overnight in an oven at 60 °C. Polymer molding was performed on the master mold using a soft silicon elastomer (Ecoflex 0030, Smooth-On, Inc.). The prepolymer was mixed with the monomer and curing agent in a 1:1 ratio in a master mold, followed by degassing and curing at room temperature for 4 h. The cured polymer was separated from the master mold and treated with oxygen plasma for 5 min to reduce surface energy. Additionally, the SAM solution ((tridecafluoro-1,1,2,2-tetrahydrooctyl)-trichlorosilane (FOTCS); Gelest Corporation, Morrisville, USA) was deposited on the patch surface for 1 h at 60 °C. Ecoflex was poured into the SAM-treated mold, degassed, and cured at room temperature for 4 h.

#### Fabrication of s-SC

A facile contact printing method was used to coat a softened double layer on the skin contact area of the facile contact printing method was utilized. First, an MED-6342 prepolymer was spin-coated onto a silicon wafer for 1 min at 500 r min^−1^ using a spin coater (SPIN-1200D, Midas Systems, Korea). The previously fabricated OIA-patterned adhesive was then transferred onto a thin liquid film formed via spin coating. No external pressure was applied during the inking step for 10 s. Then, the inked adhesive part was placed onto a clean polytetrafluoroethylene substrate with the SC facing downward to ensure the formation of a uniform thin layer on the architecture and baked for 1 h at 135 °C. After curing and demolding, a soft bilayer was coated onto the skin contact area of the SC. Finite element analysis (FEM) simulation was performed for 3D modeling of a s-SC with protuberances.

#### Cell Line

Murine fibroblast cell line NIH/3T3 was purchased from the American Type Culture Collection (Manassas, VA, USA). NIH/3T3 cells were cultured in RPMI-1640 medium containing 10% FBS (Atlas Biologicals, Colorado, USA) and 1% antibiotic–antimycotic (Welgene, Korea). Primary human mesenchymal stem cell (MSC) and human follicle dermal papillary cell (DFP) were obtained from PromoCell (Danvers, MA, USA). MSCs were cultured in Dulbecco’s modified Eagle’s medium containing 10% FBS (Atlas Biologicals, Colorado, USA) and 1% antibiotic–antimycotic solution (Welgene). DFP cells were cultured in DFP growth medium. Cells at passages 3–6 were used for all the experiments. For all in vitro experiments, cells were cultivated at 37 °C in a humidified incubator with 5% CO_2_.

#### EV Isolation

EVs were isolated from the serum-free medium conditioned by MSCs via serial centrifugation and filtration, as described previously [28]. When the MSCs reached 80% confluence of the culture T-175 flask, the 10% FBS-containing medium was removed, washed twice with DPBS, and replaced with Serum-free medium for 24 h. Collected medium was centrifuged (20 min, 3000 rpm) to deplete cell debris and filtrated through a 0.22 µm membrane to remove the cell fragments. The MSC-EVs were isolated using a tangential flow filtration capsule (Pall Corporation, Port Washington, NY, USA) and dispersed in PBS. The solution was then centrifuged twice (4000 × g, 10 min, 4 °C) for concentration, using 30 kDa Amicon tubes.

#### Preparation of Fluorescence-Labeled EV and HA

For the fluorescent labeling of EV, 8 µL Flamma 675 NHS ester (5 mg mL^−1^) was added to 1 mL EV (5 × 10^10^ particles) solution and incubated at 4 °C for 8 h. Then, the solution was purified using a PD-10 desalting column (GE Healthcare, Chicago, USA) to remove free dye molecules.

For the fluorescent labeling of HA, the labeling was carried out through conjugation of the hydroxyl group on HA with a fluorescence dye. 100 mg hyaluronic acid (10 kDa) was dissolved in 5 mL of DMSO/water (1:1) mixture. Then, 1 mg of fluorescein isothiocyanate (248–207-7, Merck, Frankfurter Strasse 250 Germany) was added. The mixture was then stirred at room temperature for 24 h before purified by dialysis against water for 24 h with membrane cutoff 3.5 kDa (Spectrum Lab, 131 Steuart Street, San Francisco, USA). The product was then freeze-dried and stored at 4 °C for further use.

#### Fabrication of MN@EV

To prepare MN@EV, a 10 wt% hyaluronic acid (HA) solution was prepared and mixed with EVs. The mixture was vortexed for 5 min and centrifuged at 3000 r min^−1^ for 10 min. The result solution (0 0.72 µL mm^−2^) was then poured into the microneedle master mold (Micropoint Technologies Pte Ltd, Singapore). Degassing was performed, and the mold was cured at 37 °C for 6 h to complete the MN@EV fabrication.

#### Fabrication of MN@EV/SC

The d-SC was treated with O₂ plasma for 1 min to enhance its bonding affinity with HA. Subsequently, the EV-loaded HA solution was cast into a MN master mold. The plasma-treated surface of the d-SC was positioned onto the filled mold to ensure direct contact and then cured at 37 °C for 6 h, followed by demolding to obtain the final MN@EV/SC patch.

#### Acetylcholinesterase (AchE) Assay

The Ampelite Colorimetric Acetylcholinesterase Assay Kit (AAT Bioquest, Sunnyvale, CA, USA) was used to measure the AchE concentration in EV. In detail, 3 × 10^8^ EVs were added into 96-well plates and mixed with 50 μL of the working solution. Samples were incubated in the dark for 30 min at 25 °C. The absorbance was collected by a microplate reader at a wavelength of 410 nm. The enzyme concentration was calculated based on the standard curve obtained by standard solutions (0–100 mU mL^−1^).

### Adhesion and Penetration Performance Evaluation

#### Analysis of Adhesion Performance on the Porcine Skin

Porcine skin sourced from a local butcher in Jidong, Korea, was used on the same day to prevent deformation. Adhesion performance was measured at 25 °C using a custom-built device (Neo-Plus, Korea) (Fig. S2). The samples were secured to a jig connected to a force sensor and brought in contact with the porcine skin. A preload of 10 kPa was applied to attach the samples to the substrates, which were detached after 5 s for measurement. The adhesion performance on the substrate was measured at least ten times, and the average values were plotted.

#### Experimental Animals

For in vivo experiments, 5-to 6-week-old male C57BL/6 mice were purchased from Orient Bio Inc. (Seongnam, Korea) and raised for ten months under specific pathogen-free conditions. All experiments involving live animals were conducted in compliance with the relevant ethical regulations and protocols approved by the Institutional Animal Care and Use Committee (IACUC) of Sungkyunkwan University (SKKUIACUC2024-04–34-1).

#### Mouse Plasma Corticosterone Assay

Plasma corticosterone levels were measured to assess stress levels in mice according to the MN length during application. C57BL/6 mice were randomly divided into five groups (*n* = 3 mice per group) as follows: Chemical adhesive; 300 µm; 600 µm; 800 µm; Control. Samples were administered to the dorsal skin of each mouse for 30 min. All mice were euthanized by cervical dislocation. Trunk blood was collected into heparinized tubes, and the serum was collected by centrifuging the clotted blood at 3000 r min^−1^ for 20 min at 4 °C. Serum samples were diluted and measured using a corticosterone ELISA kit (ab108821; Abcam, MA, USA). The absorbance at 450 nm was measured using a microplate reader according to the manufacturer’s instructions.

#### Skin Penetration Assay

Skin recovery after MN insertion was evaluated on the dorsal skin of C57BL/6 mice in vivo. Specifically, MNs of different lengths were inserted into the back skin of shaved C57BL/6 mice for 30 min. Images of the insertion area after MN insertion were obtained using a smartphone camera at 0 and 30 min after insertion.

### Transdermal Delivery Analysis of MN@EV/SC

#### Analysis of Rhodamine B Delivery Depth on Porcine Skin

Rhodamine B was dissolved in the HA solution used to fabricate MNs, enabling the detection of fluorescent signals (HA: Rhodamine B = 10:1). To observe the improvement in the delivery depth, analyses were performed while adjusting the MN length, application method, and time. Rhodamine B in the porcine skin was observed using a confocal laser scanning microscopy (CLSM, Leica, Germany). The depth of delivery was measured at ten randomly selected sites using the ImageJ software.

#### Analysis of EV Delivery Depth on Porcine or Mouse Skin

To observe the improvement in the depth of EV delivery, fluorescently labeled EVs were applied to skin substrates (porcine skin and mouse skin) for 6 h. The experimental groups were administered the following: Topi, topical administration of EV; EV/SC, topical administration of EV followed by SC application; MN@EV, application of the EV-encapsulated MN; MN@EV/SC, application of the dual-amplified patch. All groups received a liquid HA solution (10 wt%, ~ 4.9 µL) containing 3 × 10^8^ EVs. Observations and measurements were conducted under the same conditions as in 2.4.1 using CLSM and ImageJ software.

To assess Ex vivo release kinetics of labeled EVs from MNs, quantification of EV released in the porcine skin was performed according to the following procedure: i) EV-loaded porcine skin samples were grinded in a mortar for 5 min adding 0.5 mL of RIPA buffer until a homogeneous mixture was obtained; ii) extraction of labeled EV from the homogenates was carried out by adding DMSO to the mixture, which was incubated at 4 °C for 4 h; iii) the obtained solution was clarified by centrifugation at 13000 r min^−1^ for 15 min, and the fluorescence emission intensity levels of the supernatants were measured using Thermo Scientific Varioskan LUX Multimode Microplate Reader (Thermo Fisher Scientific, Waltham, MA, USA). To exclude possible interference effects caused by endogenous species, the Flamma 675-labeled EV content in the porcine skin was determined from a standard curve after subtracting the background fluorescence intensity levels of control tissues obtained from untreated skin. The amount of EV measured in the skin was eventually corrected considering the recovery factor, defined as %R=Mm/Ms, where Ms is the total amount of EV treated in the skin, and Mm is the amount of EV measured in the skin according to the procedure described above. The release kinetics of EVs were monitored over time for each group; MN@EV with sizes of 300, 600, and 800 μm were analyzed up to 6 h, whereas MN@EV/SC were assessed up to 3 h.

#### In Vivo Biodistributions of EV

For in vivo biodistribution analysis, all groups received the same amount of fluorescently labeled EVs, 1.2 × 10^9^ particles per head. The experimental groups were administered the following: Topi, topical administration of EV; EV/SC, topical administration of EV followed by SC application; MN@EV, application of the EV-encapsulated MN; MN@EV/SC, application of the dual-amplified patch (Fig. S3). The fluorescence signals of the Flamma 675-labeled EVs were observed as a function of time after local MN@EV administration to the flanks of 6-week-old male C57BL/6 mice. Fluorescence imaging was performed using an in vivo imaging system (IVIS Lumina XR, PerkinElmer). Images were captured daily until day 7. Skin tissues were collected immediately and two days after each treatment. Harvested tissues were fixed with 4% formaldehyde solution, embedded in paraffin, and sliced into 5-µm-thick sections to monitor ex vivo fluorescence by CLSM.

### In Vitro Biological Evaluation of MN@EV

#### Cell Proliferation Assay

For all in vitro cell studies, the experimental groups were incubated with the following: NC, negative control, medium containing 2% FBS; MN, a solution of blank MN; EV, a solution of EV; MN + EV, a mixed solution of blank MN and EV; and MN@EV, a solution of MN@EV. The proliferation behavior of NIH/3T3 mouse dermal fibroblast was evaluated by analyzing Ki-67 expression using flow cytometry. 3 × 10^5^ NIH/3T3 cells were seeded in 6-well plates with RPMI-1640 medium supplemented with 10% FBS and 1% AA in a humidified 5% CO_2_-containing atmosphere at 37 °C. After 24 h, the cells were treated with each sample solution for 48 h. Next, the cells were washed twice with DPBS, trypsinized using trypsin/EDTA solution, and fixed with ice-cold methanol. After 1 h, the cells were incubated with the FITC anti-mouse Ki-67 antibody (BioLegend) at 4 °C for 30 min. The Ki-67-positive cell population was analyzed using a Guava easyCyte flow cytometer (Merck Millipore, MA, USA).

#### Migration Assay

Enhanced mobility of fibroblasts following treatment with MSC-EV was evaluated using an in vitro migration assay. NIH/3T3 cells were cultured with samples on a 6.5 mm Transwell with an 8.0 μm pore polycarbonate membrane insert (Corning, NY, USA). First, warm RPMI media containing 10% FBS was dispensed into the lower chamber of the Transwell. Then, each upper chamber was seeded with 2.5 × 10^4^ NIH/3T3 cells and treated with sample solution for 10 h. For the EV-containing groups, cells were treated with EVs at predetermined concentrations (1.5 × 10^8^ particles mL^−1^ or 3.0 × 10^8^ particles mL^−1^). After 10 h, the cells that migrated to the bottom of the Transwell filter were washed with PBS, fixed with 4% formaldehyde solution, and stained with crystal violet. Bright-field microscopy was used to count migrating cells. Statistical analyses were performed using the GraphPad Prism 6 software (GraphPad Software, Inc.).

#### Soluble Collagen Assay

NIH/3T3 cells were seeded at a density of 1 × 10^5^ cells per well and incubated for 24 h. Then, the old medium was replaced with sample-containing RPMI and incubated for 48 h. At the end of the incubation period, the collected medium samples were checked for soluble collagen using the Sircol-soluble collagen assay kit (Biocolor Ltd., Carrickfergus, UK). The absorbance at 570 nm was measured using a microplate reader according to the manufacturer’s instructions.

#### Cell Viability Assay Against Oxidative Damage

NIH/3T3 cells were seeded in a 96-well plate at a density of 1 × 10^5^ cells per well. After overnight growth, the medium was replaced with sample solution in the presence of 50 µM H_2_O_2_ for 36 h. After treatment, cells were washed with phosphate-buffered saline (PBS) and the viability of the cells against H_2_O_2-_induced cytotoxicity was evaluated using Cell Counting Kit-8 (Dojindo Molecular Technologies, MD, USA) according to the manufacturer’s instructions. Afterward, the absorbance at 450 nm was measured using a microplate reader. To further investigate the elastin generation, the experiment was performed in an identical manner to that described for the soluble collagen assay. After 48 h of treatment, the collected cell pellets were analyzed using an elastin assay kit (Biocolor Ltd., Carrickfergus, UK). According to the manufacturer’s instructions, the absorbance at 513 nm was measured using a microplate reader.

#### Intracellular Reactive Oxygen Species (ROS) Assay

Intracellular ROS level was evaluated using DCF-DA. DFP cells were seeded in a 24-well plate at a density of 1 × 10^5^ cells per well and incubated until 80% confluency. Cells were then treated with samples in the presence of 150 µM H_2_O_2_ for 6 h. After treatment, cells were washed with PBS and stained with 10 µM DCF-DA probe for 30 min. Fluorescence images were obtained using CLSM.

#### Hanging Drop Assay

DFP cells were pretreated with or without 150 µM H_2_O_2_ for 1.5 h. After centrifugation, cells were re-suspended in medium containing 2% FBS and mixed with sample solutions. 20-µL drops of the final solution were forced to aggregate in the hanging drop method on the roof of a culture dish. After 5 days of incubation, cell spheroids were observed using bright-field microscopy.

### In Vivo Assessment of MN@EV/SC

#### Histological Analysis

10-month-old male C57BL/6 mice were randomly divided into five groups: Saline, EV/SC, MN, MN@EV, and MN@EV/SC. The dorsal hair of mice was shaved 24 h before the experiment. Each sample was administered for 6 h on days 0 and 3. The EV/SC, MN@EV, and MN@EV/SC groups received the same amount of EVs, 1.2 × 10^9^ particles per head. The EV doses were chosen after considering the therapeutic efficacy of MSC-EV in various animal experiments, as reported previously. Skin tissues were collected from mice on day 14 post-treatment. To quantify the soluble collagen and MMP concentrations, 10-mg of tissue fragments from each mouse was homogenized in an equal volume of physiological saline. Hematoxylin and eosin (H&E) staining and Masson trichrome (MT) staining were performed on the sections to evaluate skin regeneration and collagen deposition. The rest tissues were fixed with 4% formaldehyde solution, embedded in paraffin, and sliced into 5 µm-thick sections. Subsequently, the tissue sections were stained and observed using a slide scanner (Axio Scan Z1, Carl Zeiss, Oberkochen, Germany). Photomicrographs of three MT segments in each group were randomly selected, and the collagen volume fraction was calculated using Image J software. Dermal thickness was calculated using the embedded ZEN 3.1 software.

#### MMP-2 ELISA Assay

To further confirm the effect of EV on MMP-2 secretion, an MMP-2 ELISA was performed using commercial ELISA kits according to the manufacturer’s protocol (ab254516, Abcam, MA, USA). Specifically, 50 µL tissue homogenates solution or standard dilutions of MMP-2 and 50 µL cocktail of capture and detector antibodies were aliquoted into a 96-well microtiter plate coated with mouse anti-MMP-2 monoclonal antibody. The plate was sealed, incubated at RT for 1 h, and washed three times with 1 × washing buffer. Aliquots of 100 µL of the color reagent 3,3′,5,5′-tetramethylbenzidine were then applied for 30 min to develop a blue color, and the reaction was stopped by adding 100 µL of stop solution. The absorbance was measured at 450 nm using a microplate reader.

#### Soluble Collagen Assay

The collected tissue homogenates were used to measure soluble collagen using a Sircol-soluble collagen assay kit (S1000, Biocolor Ltd., Carrickfergus, UK). Specifically, collagen in the tissue homogenates was extracted by acid-pepsin digestion, and the Sircol assay was performed according to the manufacturer’s protocol. Finally, the absorbance was measured at 570 nm using a microplate reader.

#### Immunohistochemistry

For immunofluorescence staining, cryosections and paraffin-embedded tissue sections were prepared as described earlier. Specifically, the paraffin sections of the skin tissues were deparaffinized, blocked, and incubated with primary antibody overnight at 4 °C. Primary antibodies included those against ER-TR7 (MA1-40076, 1:50 dilution; Invitrogen, Carlsbad, CA, USA), p21 (ab241543, 1:50 dilution; Abcam), and CDKN2A/p16INK4a (EPR24167-43, 1:100 dilution; Abcam). The sections were washed five times with PBS-T and stained with fluorophore-conjugated secondary antibodies for 2 h at room temperature. Next, the nuclei were stained with 4′,6-diamidino-2-phenylindole solution. For the analysis of ROS scavenging ability of MN@EV/SC, the deparaffinized tissue sections were stained with dihydroethidium (DHE). In short, tissue sections were washed three times with PBS and stained with 10 µM DHE at RT for 30 min. Images of the stained sections were obtained by CLSM.

### Statistical Analysis

Data were analyzed using GraphPad Prism software. Efficacy data were compared using one-way ANOVA with Tukey’s multiple comparison test and reported as multiplicity-adjusted P values. All data are presented as mean ± SD. Significant differences between the datasets are indicated as follows: *p < 0.05, **p < 0.01, ***p < 0.005, ****p < 0.001, N.S. = not significant.

## Results and Discussion

### Preparation and Characterization of MN/SC

**Fig. 2:**
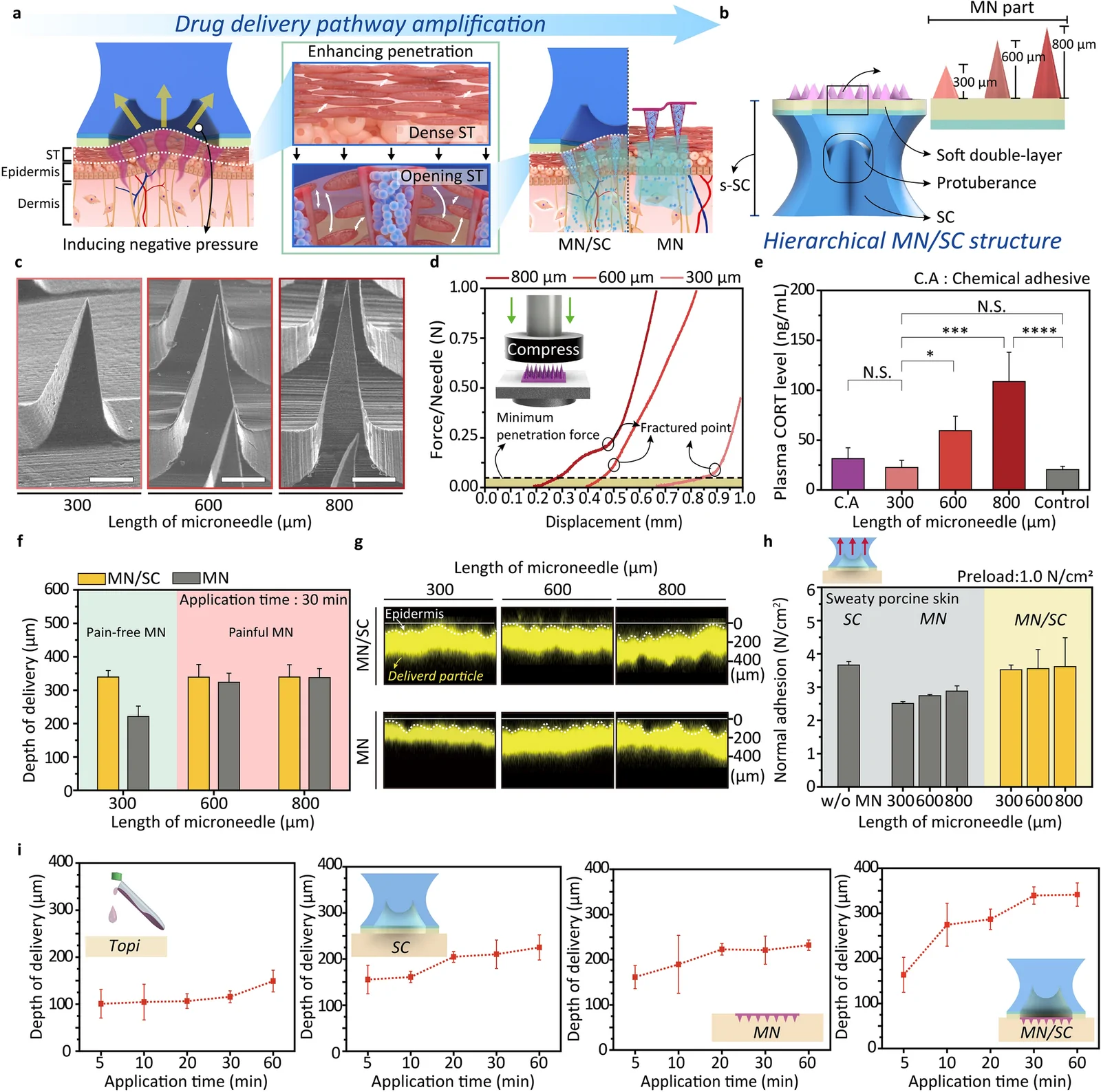
Characterization of MN/SC. **a** Schematic images showing the detailed dual-amplification mechanism through MN@EV/SC. **b** Schematic of the hierarchical MN/SC single structure showing the integration of a s-SC with MNs of varying lengths (300, 600, and 800 µm). **c** SEM images of MNs. Scale bar, 100 µm. **d** Mechanical strengths of MNs with different lengths against compressive deformation. The graph depicts the relationship between force and displacement, with the minimum penetration force marked (dashed line). The inset shows a schematic of the mechanical performance testing setup. **e** The levels of corticosterone (CORT) in the plasma of C57BL/6 mice after applying MN patches of different lengths were analyzed by ELISA (*n* = 3). **f** Delivery depth of rhodamine B into porcine skin using MN/SC and MN alone. The color of the area represents the pain level (green: pain-minimizing, red: painful). **g** Fluorescence images showing rhodamine B penetration by needle length (*n* = 10). **h** Adhesion performance in normal direction of single amplification patches (gray) and dual-amplification patches (yellow) at different microneedle lengths against sweaty porcine skin (*n* = 10). **i** Delivery depth of rhodamine B into porcine skin over time (5, 10, 20, 30, and 60 min, *n* = 10). Data are expressed as the mean ± SD. N.S. = not significant, *p < 0.05, **p < 0.01, ***p < 0.005, ****p < 0.001, by one-way ANOVA with Tukey’s post hoc test were considered (color figure online)

In order to improve the efficiency and uniformity of drug delivery to the skin, the MN/SC was prepared in this study by hierarchically combining short MNs with a bio-inspired suction cup, which mimics the structure and function of an octopus limb. Previously, we developed a suction chamber cluster patch that generates negative pressure on the skin surface and induces nanoscale deformation of the stratum corneum, thereby providing high wet adhesion performance and improving the penetration depth of drugs such as retinol (0.5 kDa) and maltol (0.3 kDa) [26]. While this approach enabled robust adhesion to wet and irregular skin surfaces without large external devices, its effectiveness was limited when delivering large molecular drugs, such as ovalbumin, resulting in poor penetration depth. This finding inspired the current study, which considers the ability to enable uniform MN application to the skin. The SC was fabricated to create a chamber with a unique protuberance structure and exert negative pressure even under moist conditions, thereby ensuring high adhesion performance (see Supplementary Notes for the theory of suction adhesion). Subsequently, the polydimethylsiloxane (PDMS) surface of SC tip was coated with the adhesives to maximize the adhesion performance (Fig. S4). Based on the adhesion behavior and drug delivery performance, the s-SC exhibited its high adaptability to curved skin surfaces. Therefore, based on the performance verification results, we adopted the structural characteristics of MN/SC as a drug delivery patch. The skin-contacting component was sequentially arranged with a MN and soft double layer. Above this layer, the SC is positioned to induce negative pressure through its structural behavior and activate additional drug delivery pathways. The MN/SC-based drug delivery process entailed two simultaneous phenomena (Fig. 2a): (1) the insertion of MN into the skin outermost layer and (2) the opening the stratum corneum facilitated by the formation of localized negative pressure, resulting in uniform MN adhesion and promoting consistent drug delivery to the dermis.

To develop a user-friendly dual-amplified transdermal delivery patch, one of the priorities was to determine the optimal MN length. Hierarchical MN/SC structures were fabricated using MNs of three different lengths (300, 600, and 800 µm) to verify their synergistic effects with s-SC (Fig. 2b). MNs of diverse lengths were spaced equally (~ 500 µm) to guarantee the same number of needles per region, and the aspect ratio was standardized (~ 3) to avoid kurtosis errors (Fig. 2c). For MN-based skin penetration, the mechanical strength of individual MNs should exceed the minimum skin invasion force (~ 0.058 N needle^−1^) [29]. To measure the mechanical behavior of individual MNs, we fabricated MN patches of identical size (~ 1 cm^2^) and compressed them with a 45-N load cell at a constant speed (0.1 mm s^−1^) (Fig. 2b, c). The experimental results showed that the MNs exhibited linearity between length and mechanical strength and that all MNs had the sufficient invasive force to penetrate the skin (Fig. 2d). To ensure that the fabrication process of MN@EV at 37 °C does not compromise EV stability, we evaluated the thermal tolerance of EVs under this condition (Fig. S5). EVs were incubated for 6 h at either 37 °C (curing condition), or 60 °C (thermal stress condition). EVs exposed to 37 °C exhibited comparable particle concentration and size to untreated controls, indicating that the fabrication temperature does not compromise EV structural integrity. In contrast, EVs exposed to 60 °C exhibited significant particle aggregation, as evidenced by a reduction in particle concentration and an increase in particle size. In further skin insertion tests, long MNs of 600 and 800 μm were shown to be completely inserted into the skin, creating a pattern of micro-wounds that remained visible even at 60 min post-application. However, the short MN (300 μm in length) exhibited inferior insertion capabilities, and micro-wounds rapidly diminished, consistent with the previous research (Fig. 1d) [30].

Next, to evaluate pain induction by MNs of different lengths, we assessed plasma CORT levels, shown to be correlated with pain response in several mouse models [31, 32]. The plasma CORT levels were monitored after the application of MNs to the back of shaved mice using a thumb press. A chemical adhesive (CA) was used as a negative control. It was worthy to note that the increase in CORT levels was dependent on the MN length (Fig. 2e). Interestingly, the plasma CORT level in mice treated with the 300-µm MNs was comparable to that observed with a mild CA that does not induce needle penetration pain. Given these results, 300 µm MNs induced negligible pain, and this observation is consistent with previous studies that quantified pain scores based on the needle length (Fig. S1).

The penetration performance of the MN/SC with different MN lengths was analyzed by observing the delivery depth of rhodamine B, a fluorescent dye chosen as the model drug. As expected, when the MNs were applied alone, the penetration depth was proportional to the MN length. Interestingly, when SC and MN were combined, the delivery depth reached an effective saturation at approximately 300 µm across all MN lengths (Figs. 2f, g, and S6). This phenomenon can be attributed to the complex relationship between the imperfect penetration efficiency of MNs and the existence of a maximum threshold in the drug delivery pathway, which is activated by the MN/SC application. In addition, the penetration ability of the MN/SC was not affected by its use in sweaty environments (Figs. 2h and S7**)**. The MN/SC provided improved adhesion performance (~ 3.5 N cm^−2^) compared to the MN-only patches of all lengths (~ 2.5, ~ 2.7, and ~ 2.8 N cm^−2^) and similar adhesion performance to that of the SC patch (~ 3.6 N cm^−2^) on the sweaty porcine skin interface (see Supplementary Notes for the theory of suction adhesion). Consequently, based on the pain response and skin adhesiveness, the 300 µm MN/SC was chosen for further experiments as the hypoallergenic drug delivery system.

Compared with other individual treatment groups (topical; Topi, SC, and MN), the performance of MN/SC was evaluated by observing rhodamine B in porcine skin using a confocal microscope as a function of time (Figs. 2i and S8). For the MN/SC group, the penetration of rhodamine B was gradually enhanced for an initial 30 min and then was maintained at a depth of 340 µm. Notably, at 30 min, the MN/SC group achieved a significantly higher delivery depth of rhodamine B than those of the other single-use groups: Topi (115 µm, ~ 34%), SC (115 µm, ~ 65%), and MN (115 µm, ~ 61%). These results suggest that the application of MN/SC induces a more significant activation of drug delivery pathways than that in the single-use groups.

### Skin Penetration Behavior of MN@EV/SC

**Fig. 3:**
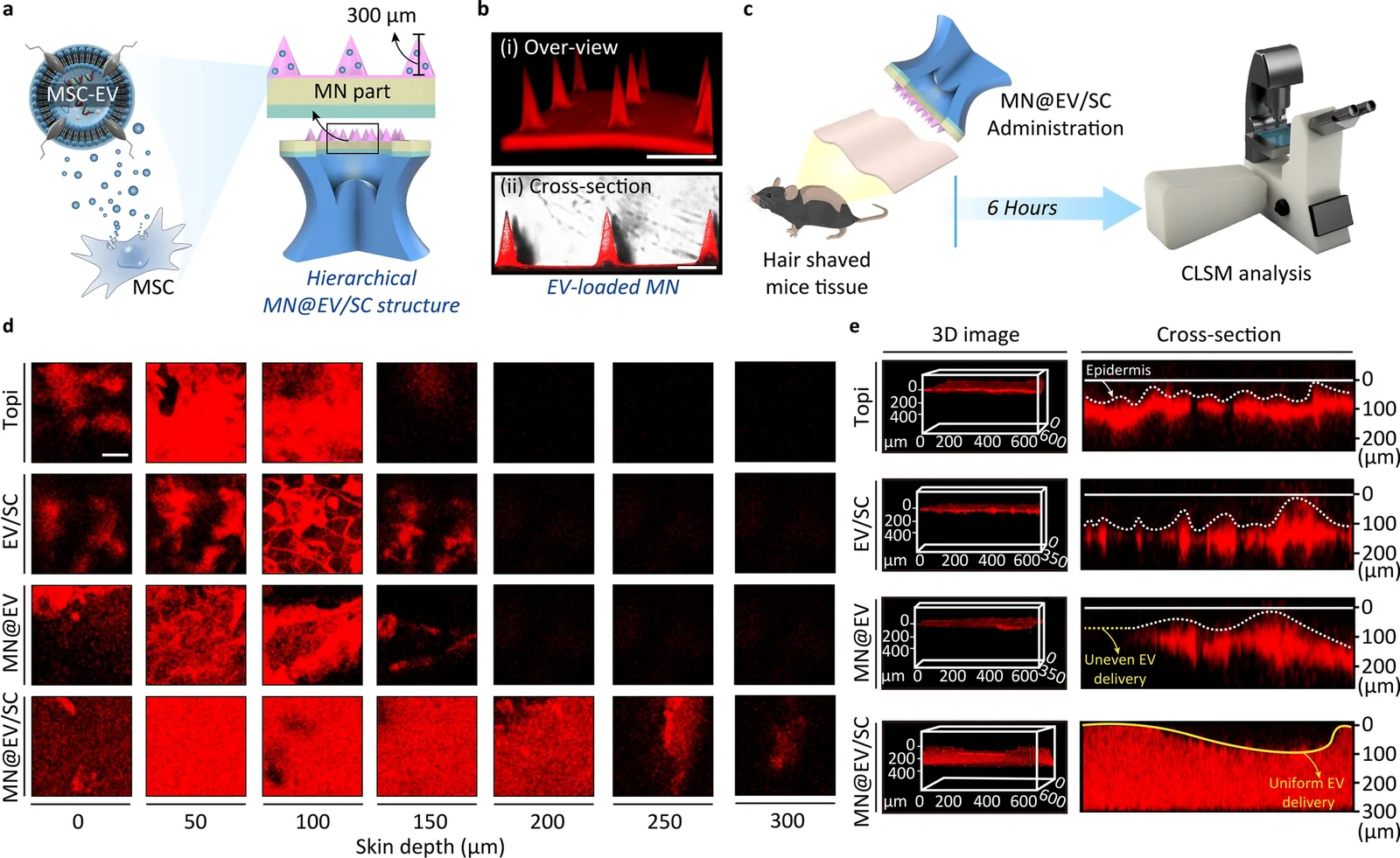
MN@EV/SC platform for efficient EV delivery. **a** Schematic of hierarchical MN@EV/SC structure. **b** 3D-reconstructed images of MN@EV captured using CLSM. Scale bar, 300 µm. **c** Schematic description and timeline for the ex vivo experiment on mice skin tissue with the MN@EV/SC. **d, e** Fluorescence images showing EV distribution across the skin depth surrounding a single suction cup of MN@EV/SC, reconstructed horizontally (**d**) and vertically (**e**) Scale bar, 50 µm

Based on the optimized structure features and adhesion properties of the MN/SC, we aimed to determine their effectiveness in delivering mesenchymal stem cell-derived extracellular vesicles (MSC-EVs). MSC-EVs have garnered attention for their potent regenerative properties to reduce inflammation, enhance angiogenesis, and even revitalize senescent cells [7, 33, 34]. These features highlight their potential in regenerative medicine, encompassing applications in wound healing, ischemic injury, and anti-aging therapies [35–38]. For effective use of MSC-EVs in regulating skin tissue, penetration through the epidermis and reaching the dermis is necessary, where they can deliver their bioactive cargo to dermal cells [39]. The epidermal layer, which is approximately 50–120 μm thick [40], restricts the penetration of EV treatments, limiting their efficacy. While the transdermal injections via syringes represent the common approach to overcome insufficient penetration into the deep dermis [41], they often result in the formation of bumps and localized tissue trauma [42]. To address these limitations, we employed MN/SC as a delivery system for MSC-EV. All the MSC-EVs, isolated from the serum-free medium conditioned by MSC cells, were characterized to determine size distribution, morphology, and surface marker expression (Fig. S9). As anticipated, the EV demonstrated nanoscale dimensions with a diameter range of 50–160 nm (average size of 138.4 nm) and a typical round spheroidal shape. Western blotting confirmed the presence of EV-specific markers (CD9 and Alix) while showing no detectable signal for the non-EV feature (calnexin), thereby verifying the successful extraction of MSC-EVs. To fabricate MN@EV, MSC-EVs were mixed with an aqueous solution of HA, a biocompatible polymer commonly used in dermatological applications, which preserves the integrity of EVs during both formulation and delivery. Then, the mixture was homogenized through vortexing and centrifugation, introduced into the MN molds for shaping, and being dried by airflow (Figs. 3a and S10). CLSM imaging using Flamma 675-labeled EV indicated successful encapsulation and even distribution of EV within the MN, confirming the reliability of the MN@EV fabrication process (Fig. 3b, c). In addition, the dual-amplified patch (MN@EV/SC), which was developed based on MN@EV, demonstrated comparable adhesive performance to MN/SC while providing both conformal attachment and high conformability to curved skin surfaces (Fig. S11).

To evaluate the capacity of the dual-amplified patch for EV delivery, ex vivo experiments with skin tissues (i.e., porcine and mouse skins) were performed. The Flamma 675-labeled EVs (3 × 10^8^ particles per tissue) were applied to the harvested skin tissue of mouse with treatments assigned as follows (Fig. 3d): topical administration of MSC-EV (Topi), SC patch adaptation after topical administration (EV/SC), EV-loaded microneedle patch without SC (MN@EV), and dual-amplified patch with MN and SC (MN@EV/SC). In contrast to the trend observed with rhodamine B, both EV/SC and MN@EV showed limited penetration ability, with penetration depths comparable to those of Topi (Fig. 3e). This discrepancy highlights the distinct biophysical behavior of EVs, whose larger size and membrane complexity hinder passive and MN-assisted diffusion. In practice, microneedles shorter than 400 μm often exhibit limited efficiency in delivering macromolecular therapeutics [43, 44]. In addition, the SC-based negative pressure mechanism is inherently less effective for larger molecules, as delivery efficiency is inversely correlated with molecular size. These limitations were particularly evident in the delivery of EVs, resulting in negligible differences in penetration depth compared to Topi. Meanwhile, the MN@EV/SC group achieved a remarkable uniform penetration depth of approximately 290 µm compared to the other single-use groups, Topi (81 µm, ~ 35%), SC (115 µm, ~ 39%), and MN@EV (111 µm, ~ 38%). The enhanced delivery depth observed in porcine skin, which closely resembles human skin, demonstrates that the dual-amplified system enables effective penetration without relying on longer MNs typically required (Fig. S12). In addition to measuring penetration depth, the EV release profile was also assessed. The MN@EV/SC group exhibited a faster release rate and achieved a comparable cumulative EV delivery to that of longer MNs (600–800 µm), highlighting the contribution of suction-based amplification to enhanced transdermal delivery efficiency (Fig. S13). Given its ability to deliver the nano-sized particles into the dermis layer, the dual-amplified transdermal patch of this study would have the potential to enhance therapeutic efficacy of valuable therapeutics such as proteins, nucleic acids, and even EVs. In addition, by reducing their doses, this patch may substantially decrease the medical expense.

**Fig. 4:**
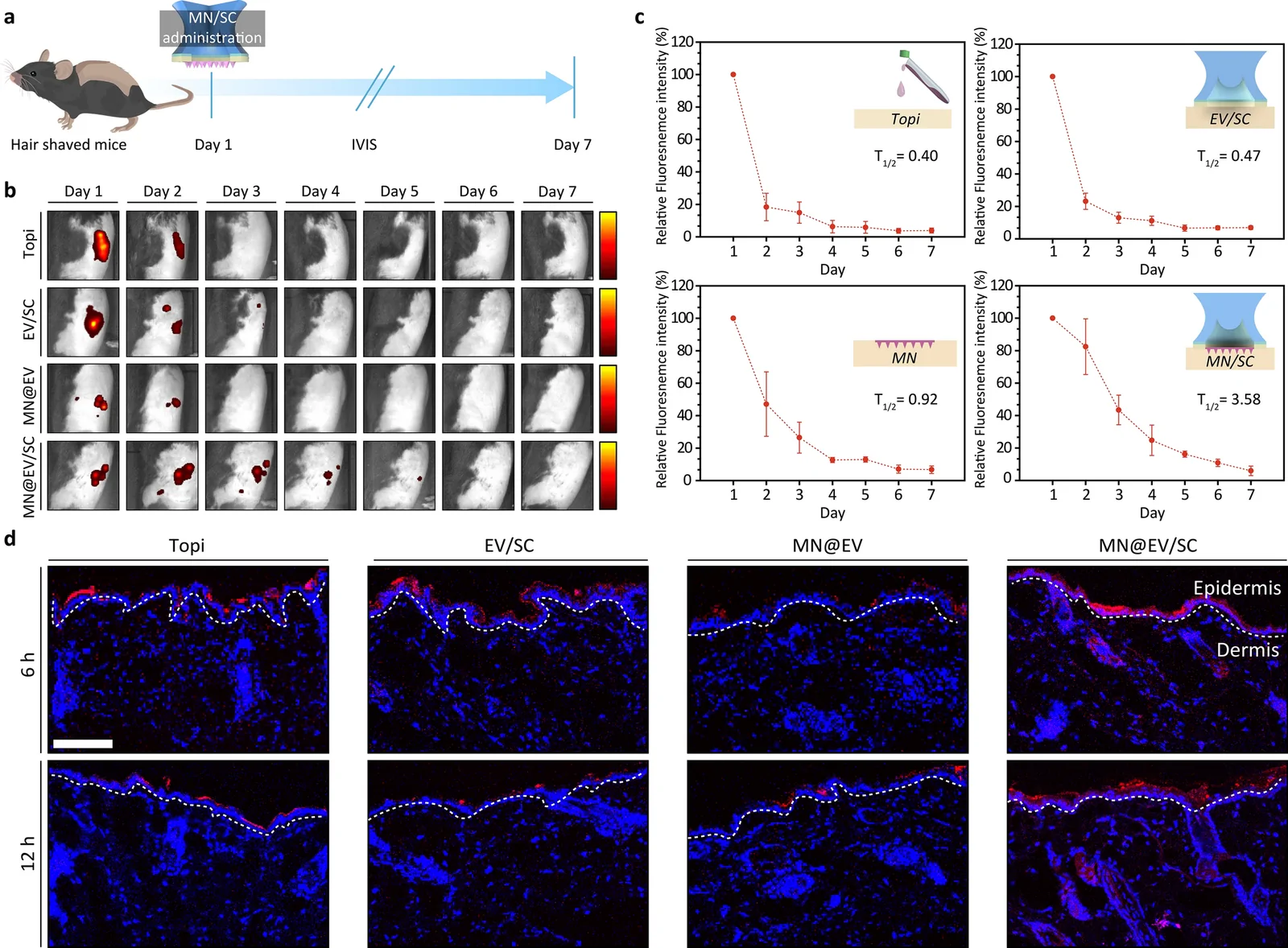
Evaluation of in vivo effectiveness and release profile of EVs via MN@EV/SC. **a** Schematic representation and timeline for the in vivo biodistribution analysis in C57BL/6 mice. **b** Representative in vivo fluorescence imaging showing EV retention over time (Day 1–Day 7) post-application (*n* = 3). **c** Quantitative analysis of fluorescence intensity across different delivery methods over time. **d** Representative fluorescence images of skin cryostat sections showing distribution of labeled EV in skin layers (epidermis, dermis, and subcutis). The white line indicates the boundary line between the epidermis and dermis. Scale bar, 100 µm

To evaluate in vivo delivery efficiency of the MN@EV/SC, fluorescently labeled EVs were monitored by determining real-time fluorescence signals using an optical imaging system in a mouse model (Fig. 4a). The labeled EVs (1.2 × 10^9^ particles per mouse) were applied to the left side of the shaved mouse back with a thumb press. Of four different treatments, MN@EV/SC showed prolonged fluorescence with signals detectable for up to five days after application, indicating the sustained release of EV into the dermal region (Fig. 4b). In contrast, both EV/SC and MN@EV showed a loss of fluorescence by day 2. The relative fluorescence intensity over time suggested that the fluorescence half-lives of MN@EV/SC and MN@EV were 3.58 and 0.92 days, respectively (Fig. 4c). Further, according to fluorescence imaging of skin cryosections, the MN@EV/SC group exhibited stronger fluorescence signals in the epidermis. Notably, EVs in this group were detected in the dermis even at a depth of 290 μm, highlighting enhanced penetration and retention of EVs compared to other groups (Figs. 4d and S14). In contrast, single MN administration did not achieve consistent penetration, which aligns with previous results. Although the MN@EV group incorporates a 10 wt% HA hydrogel that could facilitate sustained release, the limited mechanical strength of HA microneedles and the elastic properties of mouse skin likely resulted in incomplete penetration and superficial delivery. These mechanical limitations may have led to rapid signal attenuation and minimal retention of EVs in the dermis, as observed in both surface imaging and cryosection analysis. These findings are consistent with previous literature reporting that even MNs designed to penetrate hundreds of micrometers often achieve only partial insertion due to bending, buckling, or skin deformation [43, 45]. Consequently, the observed differences in EV localization and retention among groups underscore the importance of the dual-amplified mechanism of MN@EV/SC, which combines physical insertion and negative pressure to enhance transdermal delivery.

These results of effective penetration and prolonged retention, observed on the dynamic in vivo surface, validate the robustness and adaptability of the MN@EV/SC system. Therefore, this system would facilitate EV interactions with major dermal cells, such as fibroblasts and hair papilla cells, which are integral to skin regeneration and repair processes.

To assess the feasibility of ambient storage for clinical use, we examined the structural and functional stability of EVs in MN@EV patches stored at room temperature for 1 and 7 days. EVs were extracted and analyzed using NTA and AchE activity assays (Fig. S16). Both particle integrity and enzymatic activity were maintained up to 7 days, showing no significant decline compared to freshly prepared EVs. This stabilization is likely attributable to the protective effect of HA, which has been previously reported to mitigate EV degradation under non-refrigerated conditions [46]. These results suggest that the MN@EV system can be handled and stored at room temperature for short durations, adding practical value to its clinical translation.

### In Vitro Biological Functions of MN@EV

**Fig. 5:**
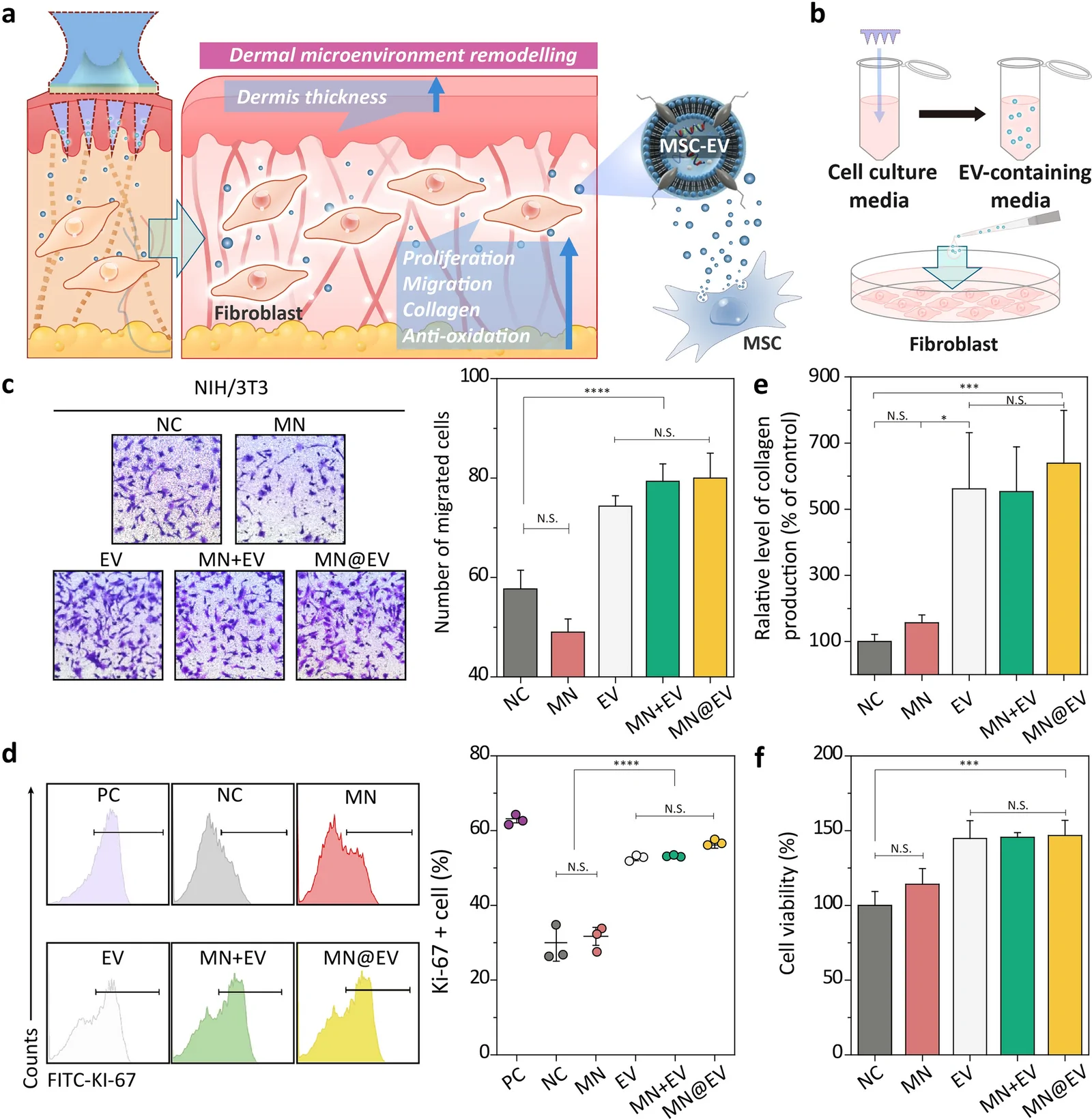
Biological function of MSC-EV loaded in MN on fibroblast. **a** Schematic of MSC-EV delivery via MN@EV and its effects on dermal fibroblast. **b** Schematic representation of EV-containing media preparation by dissolving MN@EV. **c** Representative optical microscopy images (left) and quantitative analysis (right) of NIH/3T3 cell migration in the Transwell assay. Scale bar, 1 mm. **d** Representative flow cytometry plots (left) and quantitative analysis (right) of Ki-67^+^ NIH/3T3 cells. **e** Relative soluble collagen production in NIH/3T3 cells compared to the control group. **f** Cell viability of NIH/3T3 cells under H_2_O_2_-induced oxidative stress. Data are expressed as the mean ± SD. N.S. = not significant, *p < 0.05, **p < 0.01, ***p < 0.005, and ****p < 0.001, by one-way ANOVA with Tukey’s post hoc test were considered

Recent advancements in transdermal drug delivery technologies have emphasized the prevention and rejuvenation of aged skin, a prominent and conclusive indicator of human aging [47]. Skin aging is a multifactorial process influenced by both intrinsic and extrinsic factors, including chronological aging and environmental factors such as air pollution, smoking, and ultraviolet light [48, 49]. These factors lead to a dermal microenvironment characterized by elevated oxidative stress, increased inflammation, and the accumulation of senescent dermal cells [50, 51]. Aged dermal cells lose collagen production capabilities, while increased matrix metalloproteinases and pro-inflammatory factors weaken the dermal structure and mechanical integrity [52]. Moreover, skin aging is not merely a matter of external appearances, such as pigmentation and wrinkle formation. The accumulation of senescent dermal cells renders it more sensitive and susceptible to age-related dermatological pathologies, including telangiectasia, seborrheic dermatitis, and even skin cancer [53, 54]. Consequently, amelioration of the dermal microenvironment during aging is critical for the management of additional skin diseases. Dermal fibroblasts, the primary cell type responsible for collagen production and matrix renewal within the dermis, gradually lose these capabilities with age [55]. MSC-EVs have been demonstrated to promote fibroblast proliferation, migration, and collagen synthesis, thereby enhancing fibroblast functionality and supporting skin regeneration [56] (Fig. 5a). However, EVs tend to be unstable at room temperature, which can lead to rapid degradation [57]. This raises concerns about potential losses in structural integrity and functionality during loading and storage. We aimed to ascertain whether MSC-EVs incorporated into the MN@EV system could maintain their biological activity in DFs.

To assess their ability to promote fibroblast activity, MSC-EV-loaded MNs were introduced into murine fibroblast cell line NIH/3T3 (Fig. 5b). The migration of NIH/3T3 cells was evaluated using a Transwell assay, and the number of migrated cells was quantified (Figs. 5c and S16a). The number of migration cells was enhanced in a concentration-dependent manner across all MSC-EV-treated groups. Then, the impact on proliferation was evaluated through flow cytometry for Ki-67, the primary marker utilized to assess cellular proliferation (Figs. 5d, S16b and S17) [58]. Analogous to the migration assay, Ki-67-positive NIH/3T3 cells significantly increased after incubation with MSC-EV both solution and MN form, whereas no statistically significant change was observed in the HA-only treatment group. Additionally, we assessed the collagen production in NIH/3T3 cells using a soluble collagen kit (Figs. 5e and S16c). Soluble collagen levels were markedly elevated in all EV groups, including MN-EV and MN@EV, compared to both the NC and MN groups. No significant differences were observed among the MSC-EV-treated groups. To further investigate their protective effects against oxidative stress, MSC-EVs were evaluated for their ability to prevent premature senescence and subsequent cell death in H_2_O_2_-induced fibroblasts [59]. Treatment with MSC-EV significantly enhanced the survival of NIH/3T3 cells in a concentration-dependent manner (Figs. 5f and S9d). The level of reactive oxygen species (ROS) estimated using 2′,7′-dichlorofluorescin diacetate (DCF-DA) increased in fibroblasts damaged by H_2_O_2_ exposure. After 6 h of H_2_O_2_ exposure, low concentrations of MSC-EV and MN alone provided modest post-exposure protection, whereas high concentrations completely prevented detectable ROS generation. Furthermore, MSC-EVs were found to mitigate elastin synthesis disruption caused by oxidative stress (Fig. S18). Co-treatment with selected concentrations of MSC-EV resulted in a measurable increase in elastin secretion in H_2_O_2_-induced fibroblasts, while MN alone exhibited no significant effect. These findings highlight that the therapeutic efficacy of MN@EV is primarily attributable to MSC-EV, as no discernible benefits were observed in the HA-alone group. Given the minimal HA quantity used (0.67 µg per well), its contribution to the therapeutic outcomes is likely to be negligible. Collectively, the EVs retained their inherent biological properties throughout the fabrication process of MN@EV, demonstrating outcomes comparable to those of MSC-EV in vitro. Furthermore, EVs retained their functionality despite storage at room temperature. This strongly supports the suitability of MN@EV for preservation of EV functional integrity.

Although this study primarily evaluated fibroblast activity, it was revealed that MSC-EVs not only protect fibroblasts but also enhance the resilience of hair follicle cells against oxidative stress, preserving cellular integrity (Figs. S19 and S20**)**. These results are supported by emerging evidence suggesting that MSC-EVs broadly protect diverse dermal cells from oxidative damage and maintain their functionality [60]. Additionally, suction-induced skin stretching facilitates the opening of hair follicles blocked with sebum and compression of the follicle surface toward the outer skin, thereby improving drug delivery through hair follicles [2]. Given these findings, the protective capacity of the dual-amplified patch system extends its potential applications beyond skin regeneration, such as inflammatory skin diseases, including alopecia areata, and psoriasis, where modulation of inflammatory responses and resistance to oxidative stress are critical [61].

### Effects of MN@EV/SC on Skin Microenvironment

Skin, the largest organ in the body, is a reflection of overall health status, mortality risk, and longevity [43]. Also, skin senescence propagates the aging phenotype to other tissues or organs [46]. Senescent cells remain metabolically active and exert an influence on their surrounding environment through the secretion of senescence-associated secretory phenotypes, including immune modulators, and matrix metalloproteinases (MMPs) [62]. These secretomes contribute to tissue dysfunction and manifestation of aging-associated phenotypes. In this study, the MSC-EV exhibited promising effects on dermal fibroblasts in vitro (Fig. 5), and MN@EV/SC allowed for precise delivery of EV to the dermis (Figs. 3 and 4), making it a viable candidate for enhancing dermal integrity.

**Fig. 6:**
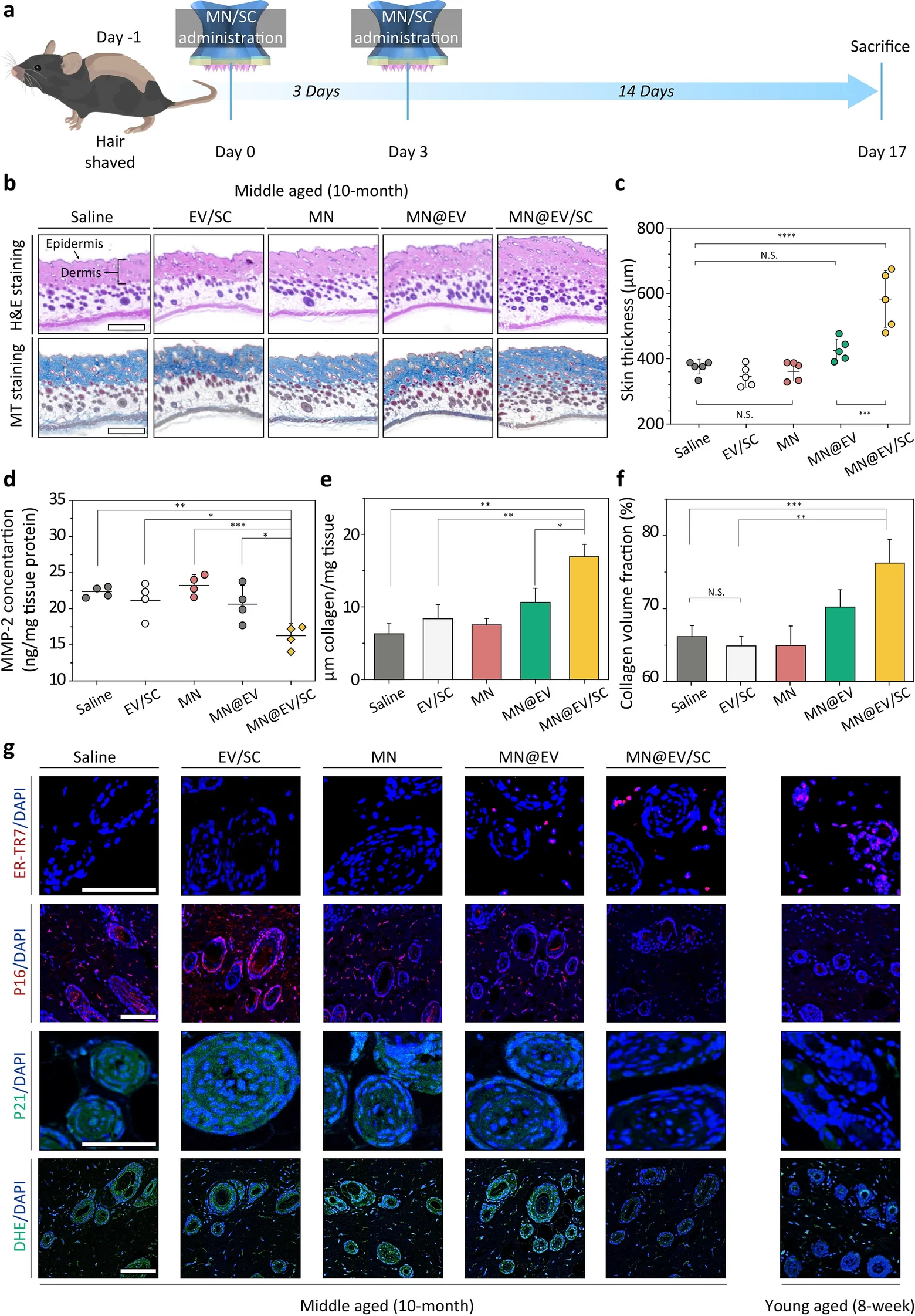
MN/SC-mediated EV delivery and improvement of the dermis microenvironment in vivo. **a** Timeline of the in vivo experiment in aged C57BL/6 mice with MN@EV/SC. **b** Histological analysis of skin tissues treated with different patches via H&E staining (upper), and MT staining (lower). Scale bar, 500 µm. Black arrows represent epidermis or dermis (*n* = 5). **c** Quantification of dermal thickness from skin sections. **d** MMP-2 concentration in mice skin homogenates. **e** Quantification of collagen-occupied area in the dermis. **f** Collagen volume fraction of skin sections. **g** Representative immunofluorescent staining images of skin tissues for ER-TR7, p16, p21, and DHE. DAPI (blue) were used for visualizing cell nuclei. Scale bar, 100 µm. Data are expressed as the mean ± SEM. N.S. = not significant, *p < 0.05, **p < 0.01, ***p < 0.005, ****p < 0.001

To evaluate the clinical potential of MN@EV/SC, we investigated whether the MN@EV/SC could promote fibroblast activity and modulate senescence markers in the dorsal skin of mice. 10-month-old C57BL/6 mice were used, which are categorized as middle-aged [63–65]. Notwithstanding their juvenile status, mice within this age bracket have been observed to manifest preliminary indications of dermal aging, including diminished skin thickness and diminished collagen production. EV/SC, MN, MN@EV, and MN@EV/SC were administered in mice twice every three days (Fig. 6a). On day 14 post-administration, skin tissues were collected for histological analysis and biochemical experiments. Skin histology revealed the effects of EV on structural changes and the amount of collagen deposition in the dorsal skin (Fig. 6b). The average thickness of the dermis increased by 563.2 ± 79.9 μm, representing a 1.6-fold increase in the MN@EV/SC group relative to the EV/SC group (Fig. 6c). In contrast, the MN@EV group exhibited no statistical differences from the EV/SC group, highlighting the superior EV delivery efficiency of the dual patch system. Quantitative analyses of MMP-2 and collagen expression in skin tissue homogenates were also carried out. Notably, MMP-2, which accumulates during aging and disassembles the extracellular matrix, was significantly reduced in the MN@EV/SC group (Fig. 6d). This reduction is likely mediated by TIMP1 in MSC-EV, which modulates MMP activity and contributes to extracellular matrix remodeling and senescence attenuation, thereby promoting extracellular matrix stabilization [66–69]. In addition, the MN@EV/SC group led to a notable elevation in collagen expression, with a 169.1% increase compared to the Saline-treated group (Fig. 6e, f). This suggests a potential anti-aging effect of MN@EV/SC through enhanced collagen regeneration while delaying collagen degradation.

The effects of MN@EV/SC on the dermal microenvironment were evaluated by immunohistochemical staining for biomarkers of fibroblasts (ER-TR7) and senescence (p16 and p21) (Figs. 6g and S21). This approach allowed for the assessment of the changes in the fibroblast phenotype and activity. The level of ER-TR7 significantly increased in the skin treated with MN@EV/SC, along with enhanced cell density, indicating that MN@EV/SC augmented fibroblast proliferation in the skin. These findings are in line with previous studies demonstrating that MSC-EVs enhance fibroblast proliferation and ECM production, ultimately leading to dermal remodeling and collagen deposition in vivo [70, 71]. In contrast, expression of p16 and p21 was reduced in fibroblasts treated with MN@EV/SC, suggesting its potential to counteract fibroblast senescence. Next, the effect of MN@EV/SC on oxidative stress, responsible for progress in the cellular aging [72], was evaluated by DHE staining of dermis tissue. Compared to MN@EV, MN@EV/SC significantly reduced the accumulation of ROS in dermal tissues. Taken together, MN@EV/SC maximized the biological potential of EV to modulate dermal fibroblast functions and demonstrated exceptional in vivo performance, particularly in delivering MSC-EV to dermal layers with high precision.

During the repeated application of MN@EV/SC, no overt indications of local irritation, erythema, ulceration, or inflammation were observed at the application sites. This observation is consistent with the established biocompatibility of HA-based microneedles and suction-based skin adhesive systems. Although specific histological toxicity evaluations were not performed in this study, the absence of local adverse reactions suggests a favorable safety profile for dermal use. Subsequent investigations, encompassing exhaustive local and systemic toxicity evaluations, are imperative to substantiate further the safety of repeated application in a clinical context.

Since cellular aging is an inevitable and sustained physiological phenomenon, repeated treatments with biologically active components for senescent cells are indispensable for maintaining the therapeutic benefits in anti-aging applications. In this regard, it should be emphasized that the dual-amplified patch could be an effective and minimally invasive delivery system to ensure sufficient dermal penetration of therapeutics while improving patient compliance. Moreover, as the largest organ in the body which holds diverse cells, skin has been considered a key target for treatments of various diseases, including keloid, psoriasis and diabetes [61, 73]. Thus, the dual-amplified patch could offer a promising platform for clinical applications beyond cosmetic skin care. Several recent studies have reported advanced transdermal delivery platforms designed to promote wound healing and tissue regeneration via microneedles, hydrogels, or combinatorial strategies [74, 75]. In line with these developments, the MN@EV/SC system enables precise and sustained dermal delivery of EVs, thereby supporting skin regeneration through modulation of the dermal microenvironment.

## Conclusions

In summary, a novel MN@EV/SC patch was developed to address critical challenges in transdermal delivery of biologics such as proteins, nucleic acids, and EVs. This system hierarchically combines short dissolving MNs (≤ 300 μm) with a bio-inspired suction chamber. Without bulky external devices, the nanoscale deformation was used to achieve robust adhesion of the MNs and efficient delivery of the EVs into the dermis, even on irregular and moist skin surfaces. This innovative approach addresses the limitations of conventional MN systems by minimizing pain and skin damage. Representatively, the MN@EV/SC patch preserved the biological activity of EV, facilitating critical regenerative processes, including fibroblast proliferation, migration, and collagen synthesis. These effects were also validated in vivo, as demonstrated by significant improvements in dermal thickness, collagen deposition, and reduced senescence markers in mouse. The findings underscore the efficacy of the MN@EV/SC system in modulating the dermal microenvironment. Despite its promising results, the current MN@EV/SC design has limitations that warrant further investigation. Since the MNs are composed of dissolvable materials, the device is currently designed for single use. As a result, reloading and repeated application require the microneedle layer to be remanufactured. In future iterations, integrating interchangeable drug modules or improving the mechanical robustness of reusable components could improve the cost-effectiveness and usability of the system. Given its unique features, including multifunctionality, patient-friendly design, and electronics-free operation, the dual-amplified patch system might be a suitable candidate for a wide range of dermatological applications.

## Supplementary Information

Below is the link to the electronic supplementary material.

Supplementary file1 (DOCX 7688 KB)
