# Supplementary material for: A Hierarchical Short Microneedle-Cupping Dual-Amplified Patch Enables Accelerated, Uniform, Pain-Free Transdermal Delivery of Extracellular Vesicles
Source: Nanomicro Lett. 2025 Jul 23;18:11. doi: 10.1007/s40820-025-01853-7 (PMC12287502; doi:10.1007/s40820-025-01853-7)
Supplement: Supplementary file 1 — Supplementary file1 (DOCX 7688 KB) [file 40820_2025_1853_MOESM1_ESM.docx]

Supporting Information for

**A Hierarchical Short Microneedle-Cupping Dual-Amplified Patch Enables Accelerated, Uniform, Pain-Free Transdermal Delivery of Extracellular Vesicles**

Minwoo Song^1,#^, Minji Ha^1,#^, Sol Shin^1,#^, Minjin Kim^1^, Soyoung Son^1^, Jihyun Lee^1^, Gui Won Hwang^1^, Jeongyun Kim^1,2^, Hieu Duong Van^1^, Jae Hyung Park^1,2,3,^*, Changhyun Pang^1,2,^*

^1^ School of Chemical Engineering, Sungkyunkwan University, 2066 Seobu-ro, Jangan-gu, Suwon 16419, Republic of Korea

^2^ Department of Health Sciences and Technology, SAIHST, Sungkyunkwan University, Seoul 06355, Republic of Korea

^3^ Biomedical Institute for Convergence at SKKU (BICS), Sungkyunkwan University, Suwon 16419, Republic of Korea

^#^ Minwoo Song, Minji Ha, and Sol Shin contributed equally to this work.

*Corresponding authors. E-mail: [jhpark1@skku.edu](mailto:jhpark1@skku.edu) (Jae Hyung Park); [chpang@skku.edu](mailto:chpang@skku.edu) (Changhyun Pang)

**S1 Experimental**

**S1.1 Fabrication of Polydopamine coated SC (dp-SC)**

XPS, Polydopamine solution was prepared by dissolving dopamine in 10 mM Tris buffer (pH 8.5). The SC was immersed in the polydopamine solution at 25 °C for 24 h, after which it was separated from the solution. The SC was then washed three times with deionized (DI) water to remove any unbound molecules, yielding dp-SC.

**S1.2** **Finite element analysis (FEM) simulation**

FEM simulation analysis was performed using commercial software (COMSOL Multiphysics version 5.2a, license number 5084832, Altsoft, South Korea) to demonstrate the stress distribution in the s-SC during attachment. A 3D model of a s-SC with protuberances was designed. The model is automatically meshed using triangular elements. The mesh sizes ranged from 0.3823 to 0.9229 mm. It was assumed that all materials had linear elastic properties and that the suction cup of the s-SC experienced stress in a compressive state.

**S2 Supplementary Note and Figures**

Adhesion analysis of the s-SC on the wet surface.


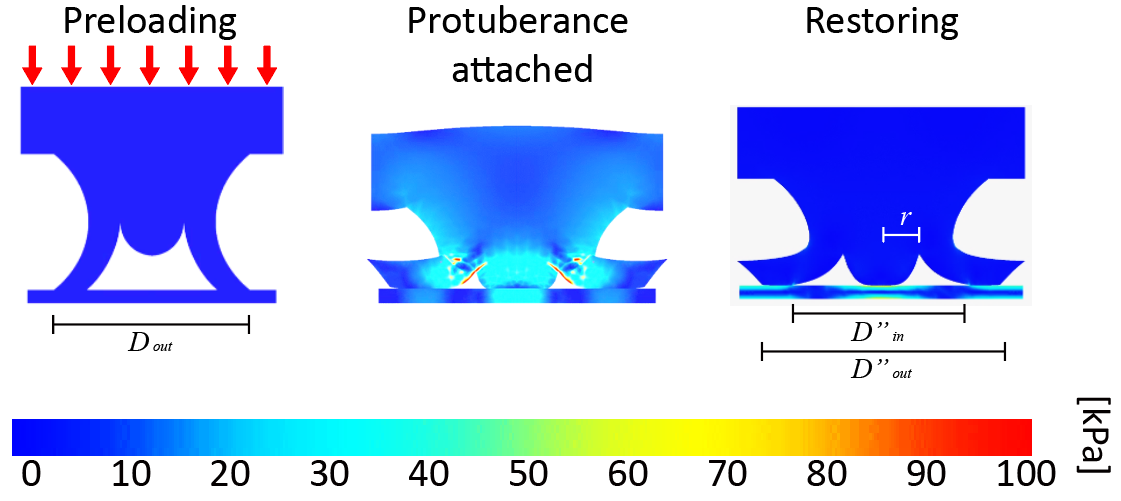


**Scheme S1** FEM simulation of deformation of the s-SC during attachment

The MN part of the MN/SC patch is fabricated based on hyaluronic acid (HA). HA is a representative biodegradable microneedle manufacturing material that is widely used [S1]. In this study, the MN/SC patch was applied to the skin in a moist environment. HA contains ammonium ions, magnesium, and alkali metals, so it has high solubility in water [S2]. Due to these properties, the backing layer of the MN is rapidly dissolved after injection. This provides conditions for the soft double-layered suction cup (s-SC) to effectively behave in a moist environment. Therefore, we theoretically analyzed the correlation between the behavior of the s-SC in a moist environment and its adhesive performance.

The preload (10 kPa) required to perform the structural function of the s-SC was applied [S3]. With the application of preload, the diameter (${D"}_{out})$ after attachment increased further in comparison to the initial diameter of the structure ($D_{out}$). As shown in figure S1, these deformations were derived based on FEM simulations.

The total wet adhesion ($\sigma_{total}$) of s-SC can be expressed by using the capillary force ($\sigma_{capillrary}$), suction force ($\sigma_{suction}$) and their actual contact surface area.

$\sigma_{total}=\sigma_{capillrary}+\sigma_{suction}$(S1)

The capillary force can be defined as the sum of the Laplace pressure and surface tension applied to a unit area.

$\sigma_{capillrary}=\left[ \frac{\pi\cdot({{D"}_{out}}^{2}-{{D"}_{in}}^{2}+4r^{2})}{4}\cdot\gamma\left( \frac{\cos\vartheta_{1}+\cos\vartheta_{2}}{h} \right)+l\cdot\gamma\right]\cdot n$ (S2)

where ${D"}_{in}$ is the inner diameter of the contact surface when a vacuum is established in the inner chamber, ${D"}_{out}$ is the outer diameter of the contact surface when a vacuum is established in the inner chamber, *r* is the radius of the dome in contact with the substrate, $\gamma$is the surface tension of the liquid, $\vartheta_{1},\vartheta_{2}$ are the contact angles on the adhesive and engaged substrate, h is the liquid gap, and l is the outer circumference, $n$ is the number of s-SCs.

Second, the suction stress of the structure under wet conditions is expressed as

$\sigma_{suction}=\left[ \frac{{P_{0}\pi{D"}_{in}}^{2}}{4} \right]\cdot n$ (S3)

where $P_{0}$ is atmospheric pressure (~ 101.3 kPa).

The total wet adhesion considering all applied forces can be summarized as follows:

$\sigma_{total}=\left[ \frac{{P_{0}\pi{D"}_{in}}^{2}}{4}+\frac{\pi({{D"}_{out}}^{2}-{{D"}_{in}}^{2}+4r^{2})}{4}\cdot\gamma\left( \frac{\cos\vartheta_{1}+\cos\vartheta_{2}}{h} \right)+l\cdot\gamma\right]\cdot n$ (S4)


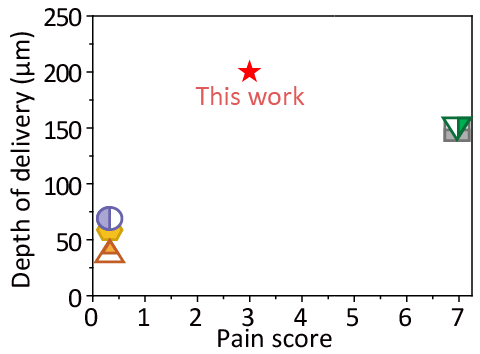


**Fig. S1** Comparison of drug delivery depth and needle length between the MN@EV/SC patch developed in this study and MN-based drug delivery systems reported in the literature


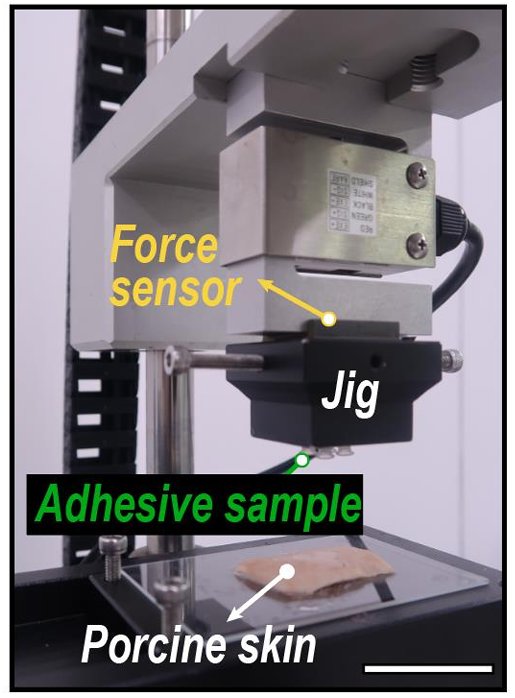


**Fig. S2 Ex vivo setup for preload-controlled adhesion test of MN@EV/SC.** Experimental apparatus for measuring the normal adhesion force of the adhesive sample using a porcine skin substrate and a load cell. A custom-built device was used to apply a preload of 10 kPa, which corresponds to a gentle force similar to that exerted by the finger


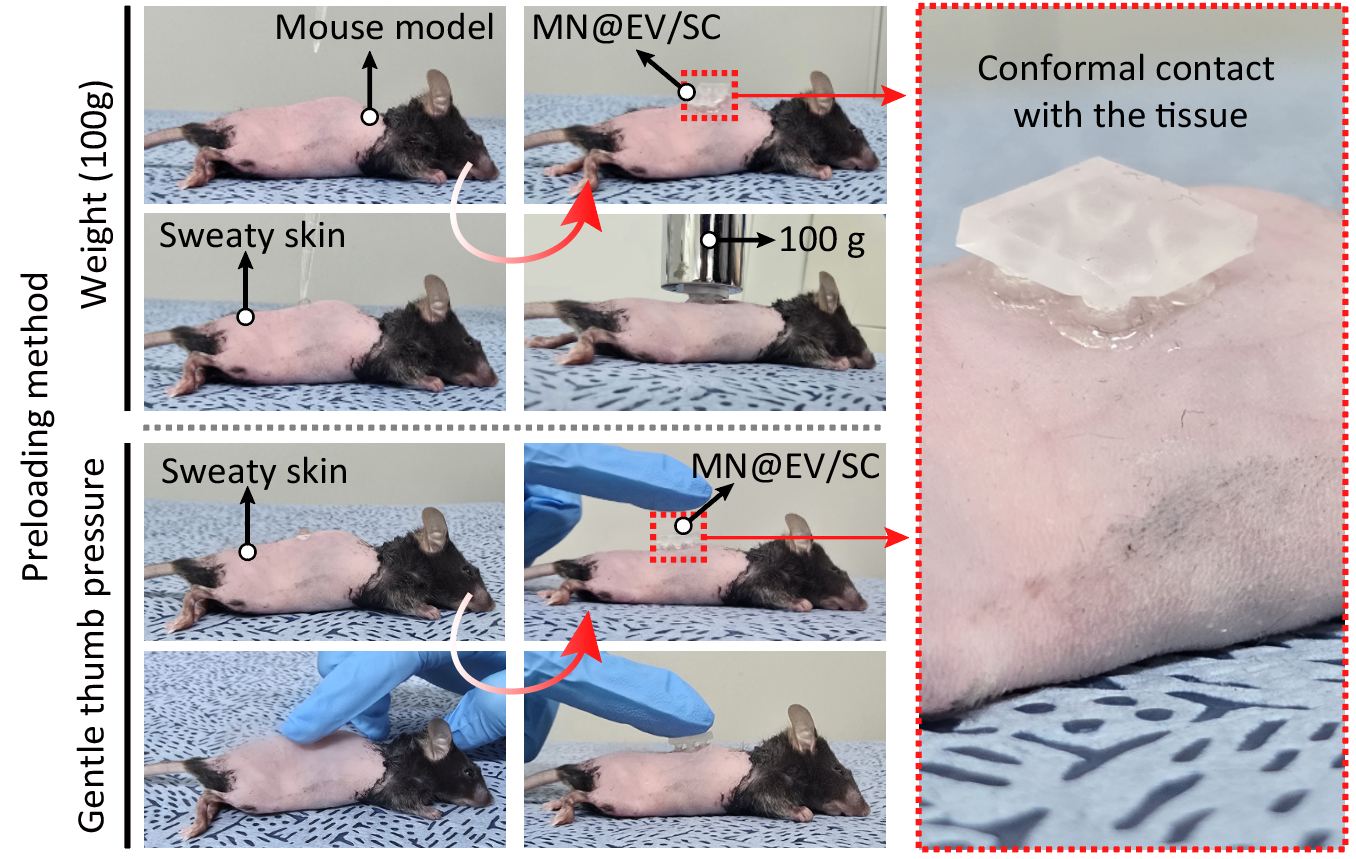


**Fig. S3 In vivo application of MN@EV/SC patch to mouse dorsal skin.** Representative photographs showing the practical application of the patch using gentle thumb pressure without additional instruments. The MN@EV/SC was stably adhered to the shaved back skin of mice, forming close conformal contact with the tissue. It confirms that the patch can be applied under real-use conditions without needing external pressure-loading devices


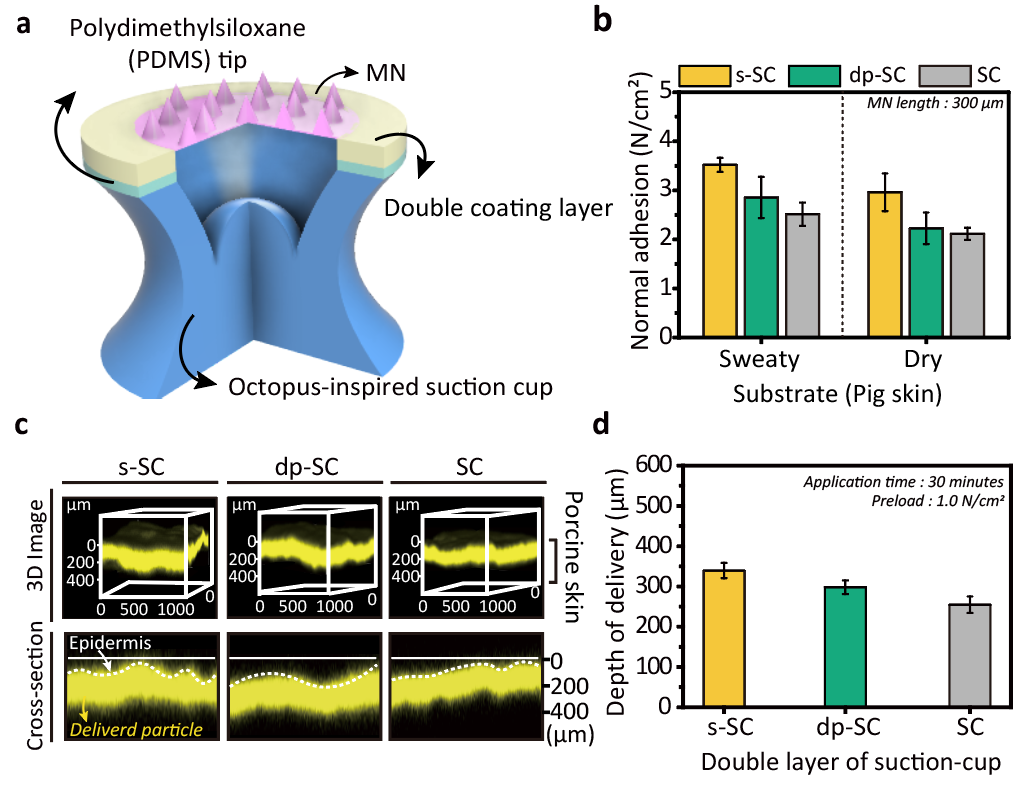


**Fig. S4 a** Schematic of the MN/SC single structure. **b** Normal adhesion performance of MN/SC depending on the presence or absence of a double-layer coating and the type of coating material (s-SC: MED-6342, dp-SC: polydopamine). **c, d** Confocal microscopy analysis of rhodamine B through porcine skin using MN/SC, depending on the presence or absence of the double-layer coating and type of coating material. Representative images (**c**) and quantification of delivery depth (**d**). The white dashed line marks the epidermis boundary


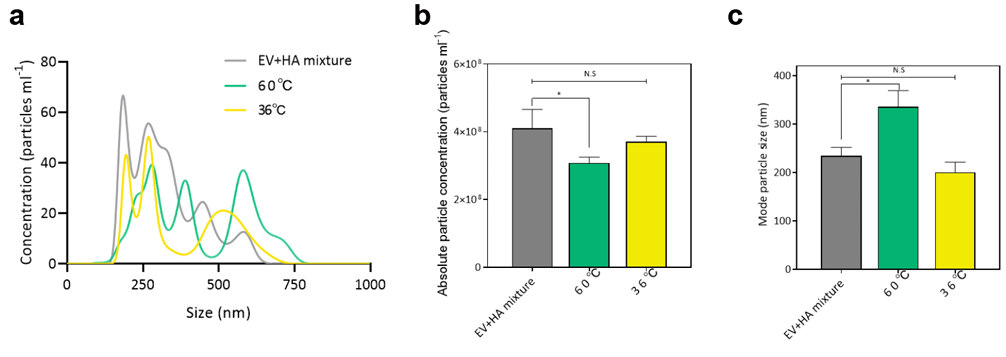


**Fig. S5 Stability assessment of EVs under thermal stress. a** Size distribution, **b** particle concentration, and **c** mode particle size of EV fabricated at 37°C or 60°C


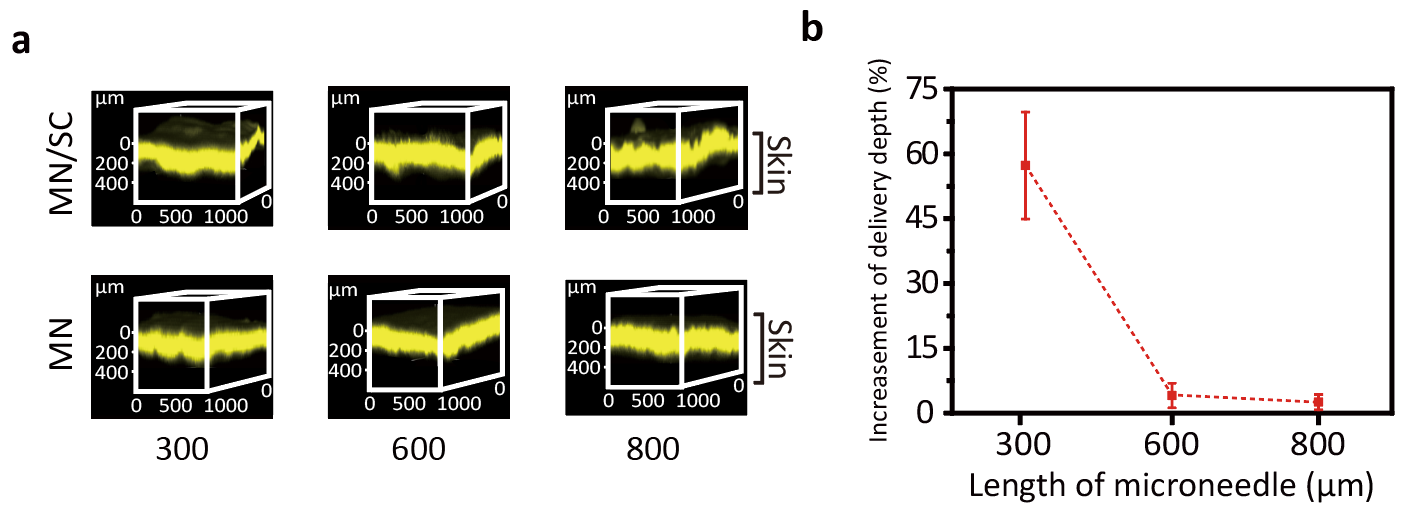


**Fig. S6** **a** Typical 3D fluorescence images showing delivery depth of rhodamine B with MN/SC. **b** Percentage increase in delivery depth achieved with MN/SC compared to MN alone as a function of microneedle length (*n* = 10)


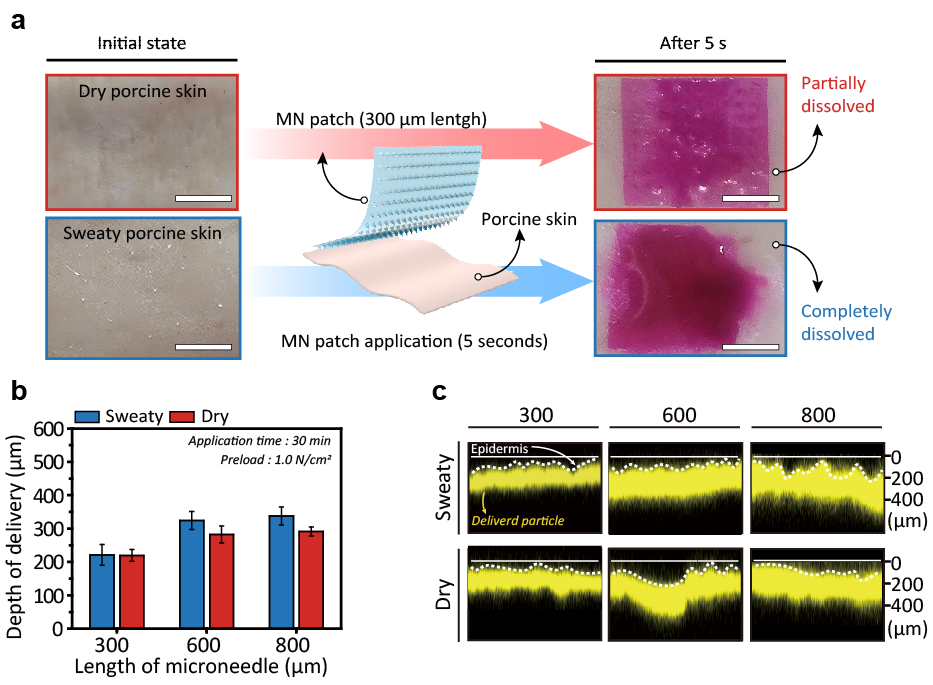


**Fig. S7** Evaluation of rhodamine B delivery using MN/SC under sweaty and dry porcine skin conditions. **a** Representative image of MN application on authentic porcine skin with dry (top) and sweaty (bottom) conditions, showing dissolution behavior 5 seconds post-application. Scale bar, 3 mm. **b** Delivery depth across microneedle lengths (*n* = 10). **c** Representative 3D fluorescence images visualizing delivery profiles. The white dashed line marks the epidermis boundary


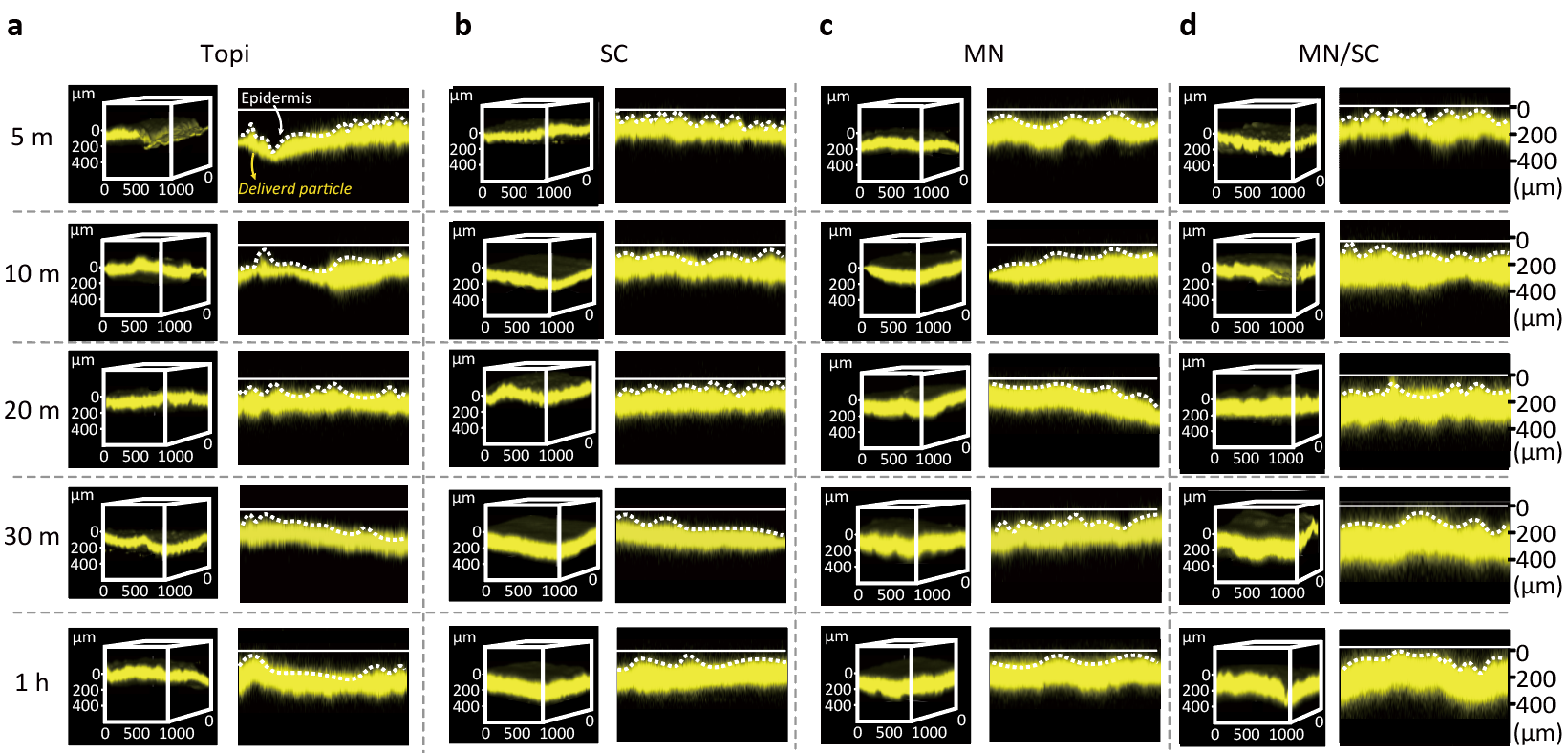


**Fig. S8** Confocal microscopy analysis of rhodamine B delivery through porcine skin using different methods: **a** Topi, **b** SC, **c** MN, and **d** MN/SC, across varying application times (5 min, 10 min, 20 min, 30 min, and 1 h). Representative 3D and cross-sectional images show the delivery profiles, with the white dashed line marking the epidermis boundary


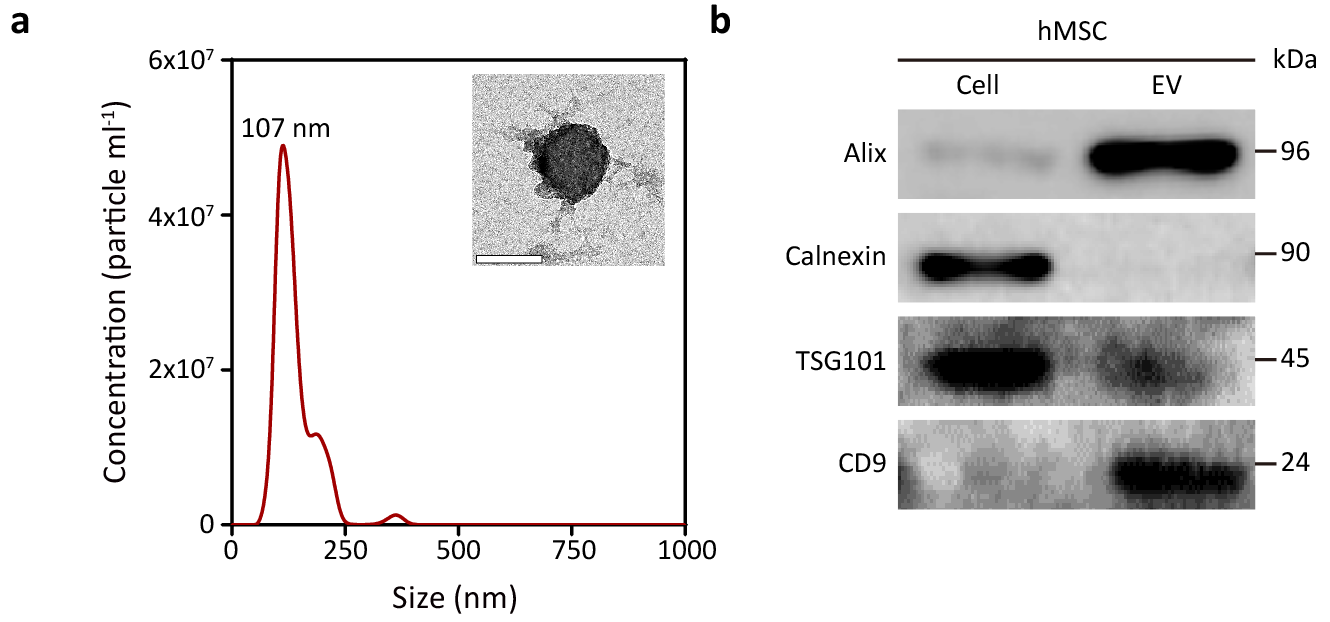


**Fig. S9** EV characterisation. **a** Size distribution of EV isolated from MSCs by NTA. The inset shows a representative TEM image of EV. Scale bar, 200 nm. **b** Western blot analysis of MSC lysate and isolated EV


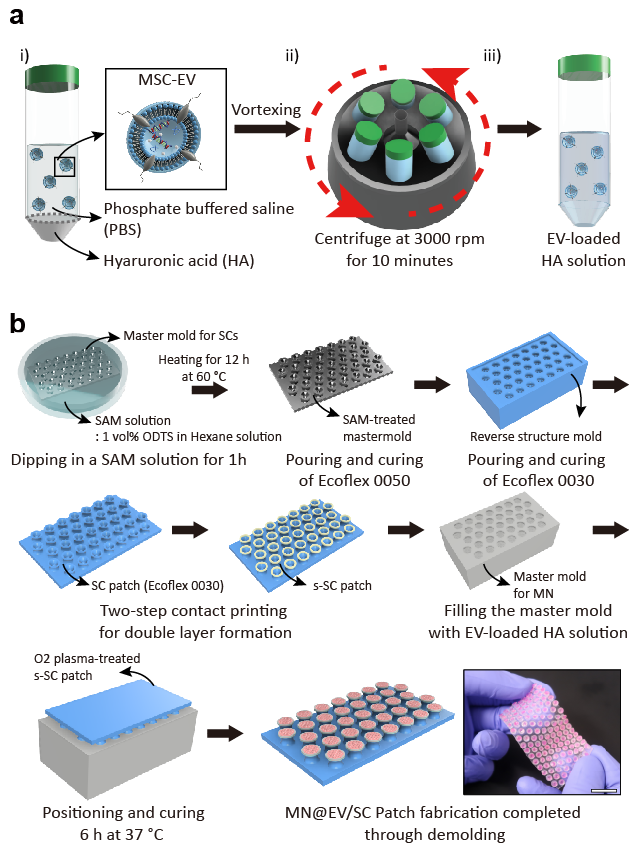


**Fig. S10 a** Schematic diagram of the preparation process for EV-loaded HA solution. i) Mixing HA with PBS containing EVs. ii) Vortexing followed by centrifugation at 3000 rpm for 10 minutes. iii) Final EV-loaded HA solution ready for MN fabrication. **b** Schematic diagram of MN@EV/SC fabrication


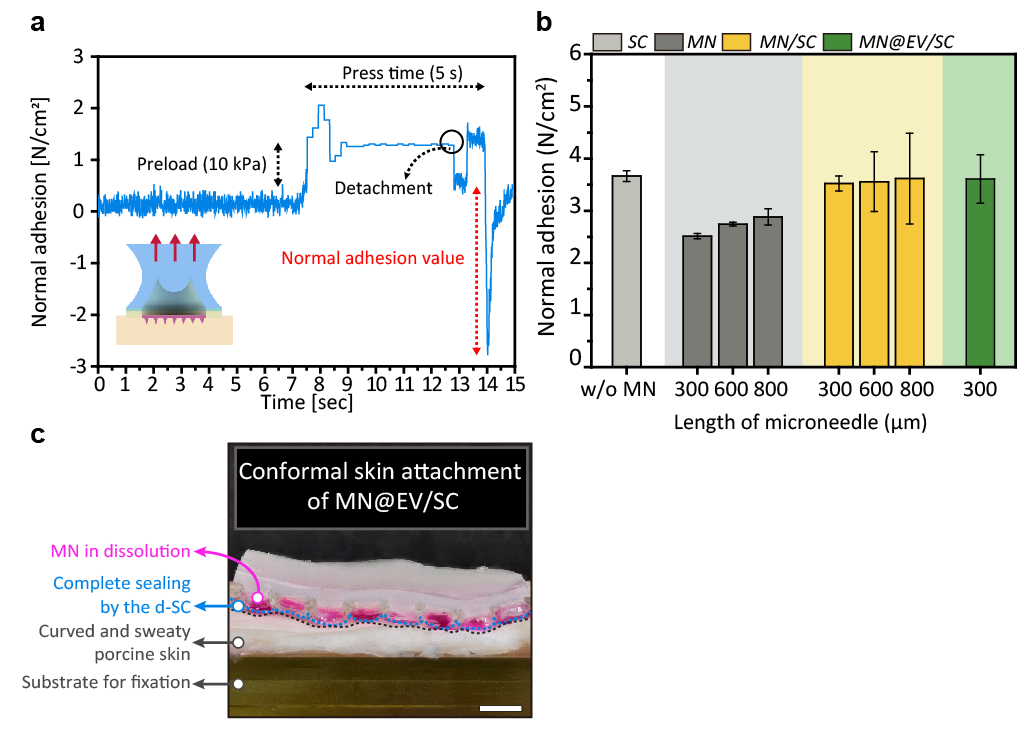


**Fig. S11 a** Representative profile of normal adhesion for the MN@EV/SC device. **b** Adhesion performance in the normal direction of single amplification patches (gray), dual amplification patches (yellow), and MN@EV/SC (green) on sweaty porcine skin at various microneedle lengths (*n* = 10). **c** Representative image of the MN@EV/SC device attached to curved and sweaty authentic porcine skin. Scale bar, 3 mm


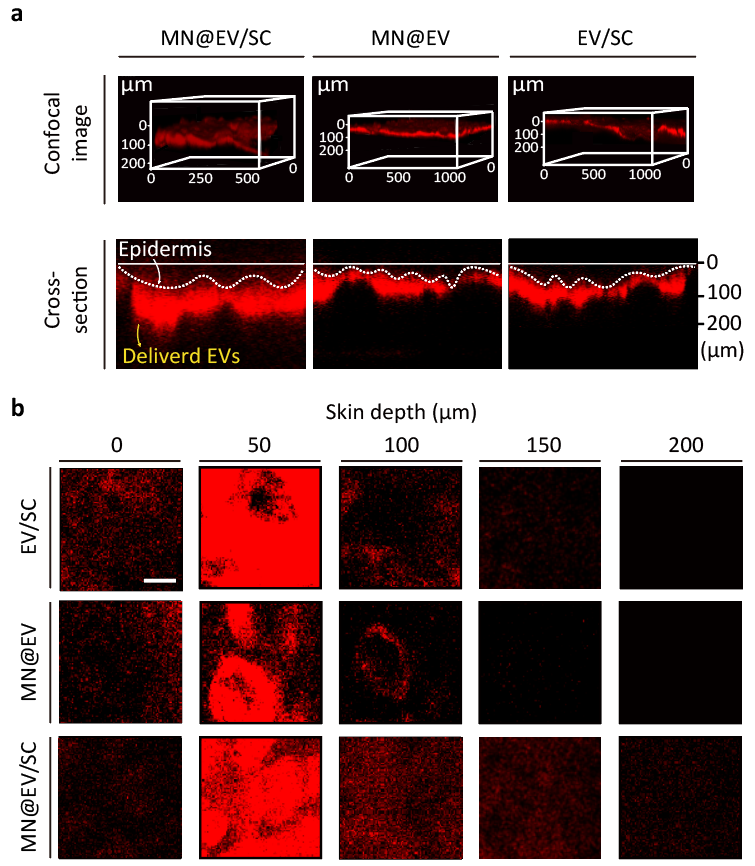


**Fig. S12** Delivery depth of EV into porcine skin using various delivery methods. **a** The representative 3D image was reconstructed based on the images obtained at various depths. **b** Fluorescence signal intensity distribution of EVs at various skin depths (0–200 μm). Scale bar, 50 μm


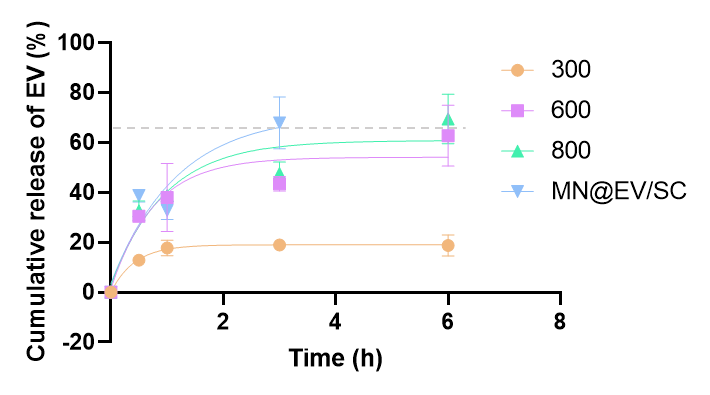


**Fig. S13** Ex vivo release kinetics of labeled EVs from MNs on porcine skin. Quantification of EV release at designated time points


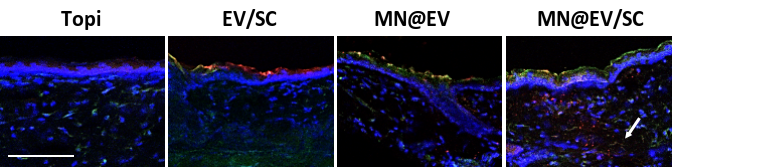


**Fig. S14** Fluorescence images of cross-sections of mouse skin after application of FITC-labeled HA MNs loaded with Flamma-labeled EV for 6 h. Blue indicates DAPI-stained nuclei, green represents FITC-labeled HA, and red corresponds to Flamma-labeled EVs. Scale bar, 100 μm


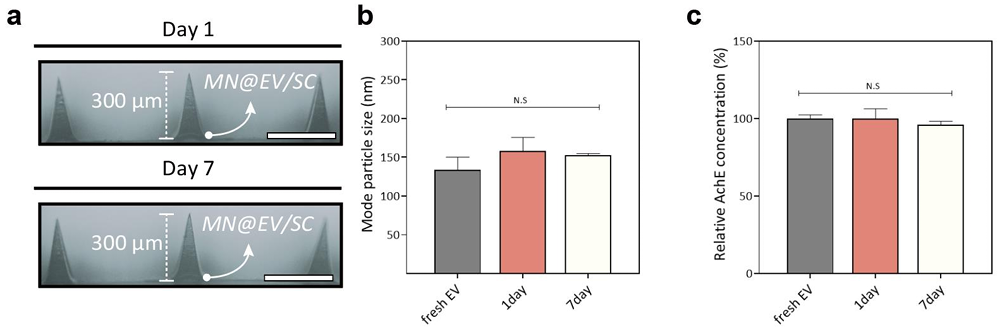


**Fig. S15 Storage stability of MN@EVs at ambient temperature. a** Representative photography of MNs. **b** Mode particle size, and **b** AchE concentration of EVs extracted from MN patches stored at room temperature for 0, 1, and 7 days


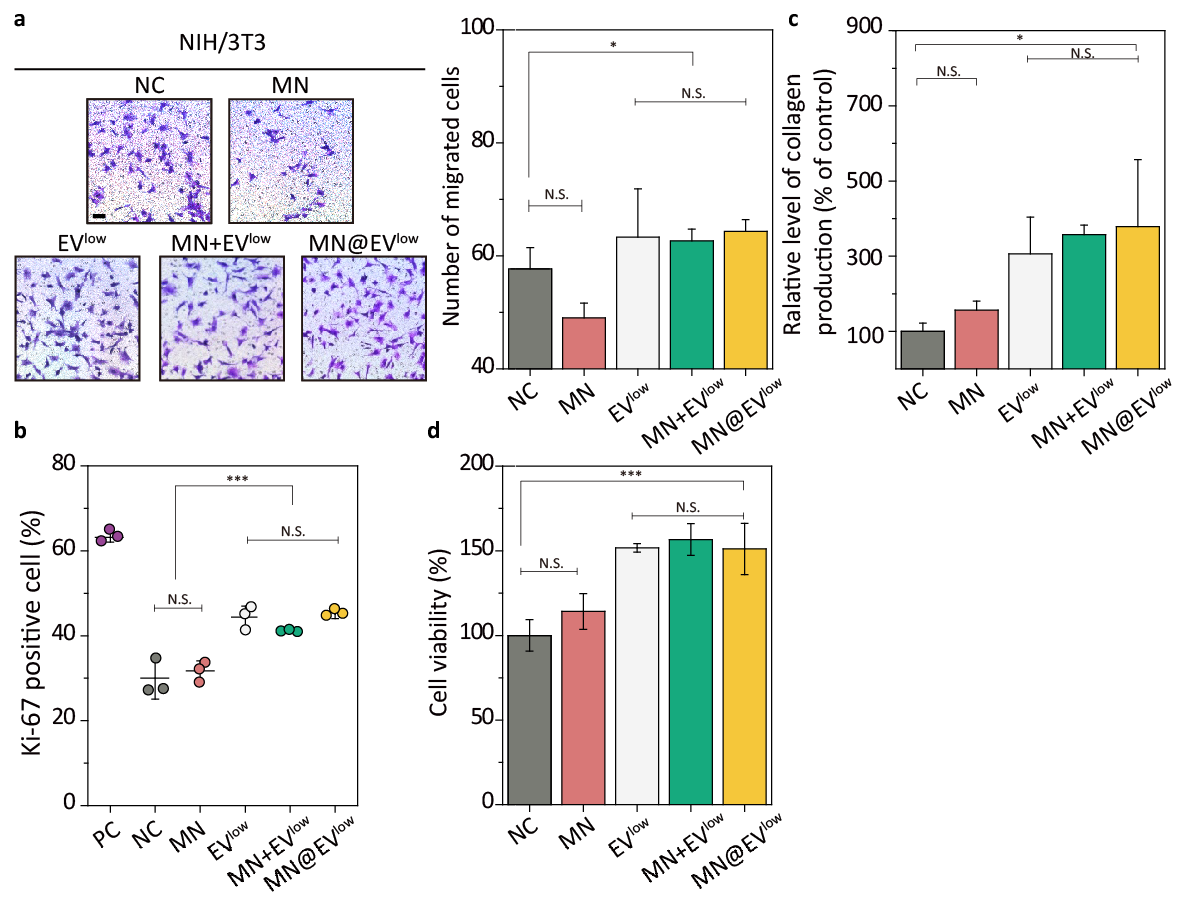


**Fig. S16** Biological functions of MSC-EV loaded in MN on dermal fibroblast. **a** Transwell assay to assess the effect of MSC-EV on the migration potential of fibroblasts. Scale bar, 100 μm. The cells in each group were treated with following groups: HA only (MN), MSC-EV^low^, MSC-EV mixed with HA (MN+EV^low^), and HA needle-loaded MSC-EV (MN@EV^low^). EV concentration of 1.5 × 10^8^ particles mL^-1^ was used for the EV-containing groups. Representative optical microscopy images (left) and quantitative analysis (right) of NIH/3T3 cells (upper chamber) using crystal violet staining. Scale, 1 mm. **b** Proportion of Ki67^+^ NIH/3T3 cells. **c** Collagen production analysis based on soluble collagen assay. Relative collagen concentration in the cell supernatant compared to the control group. **d** Cell viability of H_2_O_2_ oxidative stress induced NIH/3T3 cells of each group compared to the control group


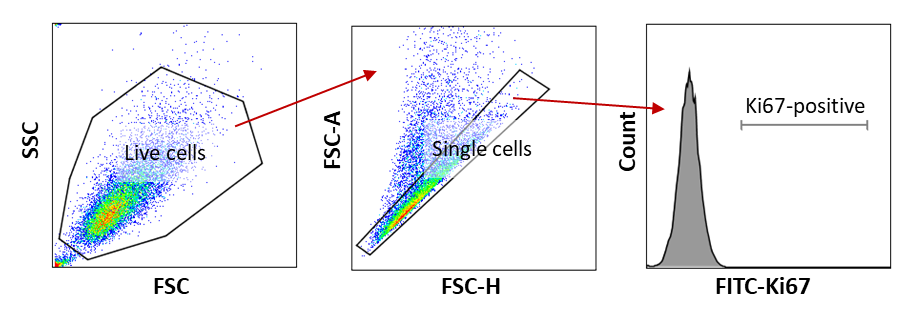


**Fig. S17** Flow cytometry analysis of fibroblasts. **a** Representative gating strategy for fibroblasts. Live cells were quantified by area and antibody labelling. CSFE-labelled cells were analyzed inside gate. **b** Representative gating strategy for splenocytes. Live cells were quantified by area and antibody labelling. Ki67-labelled cells were analyzed inside the gate


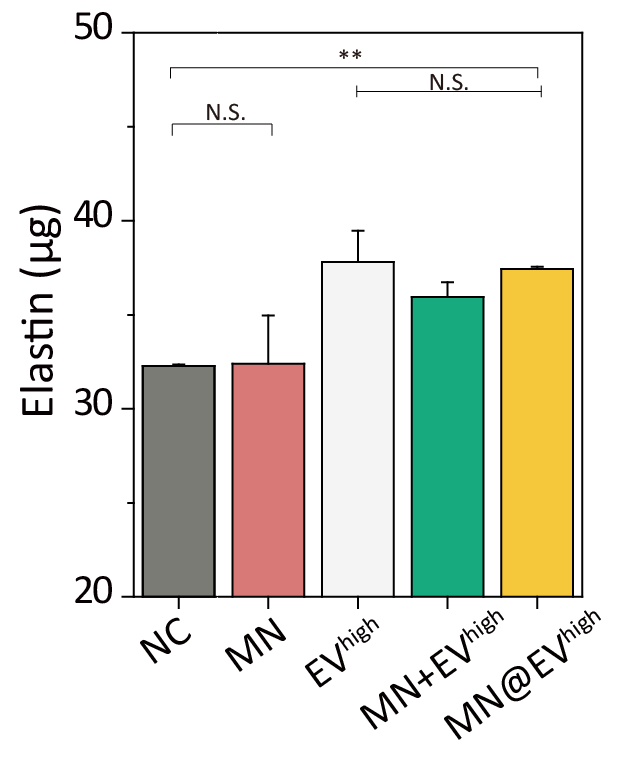


**Fig. S18** Elastin expression levels measured in the cells compared to the control group. EV concentration of 3.0 × 10^8^ particles mL^-1^ was used for the EV-containing groups


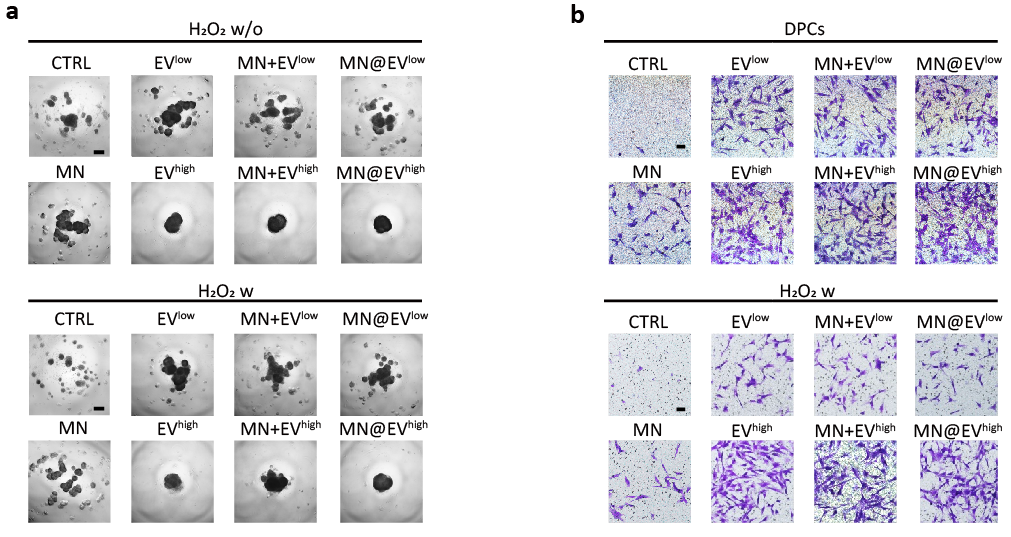


**Fig. S19** Migration and hair follicle induction abilities of EV-treated DFP cells. DFP cells were treated samples under 150 μM H_2_O_2_ (H_2_O_2_ w) or H_2_O_2_-free (H_2_O_2_ w/o) conditions. **a** Self-aggregation of DFP cells in hanging drops. **b** Representative images of migrated DFP cells stained with crystal violet. Images were captured using CLMS. Scale bar, 100 µm


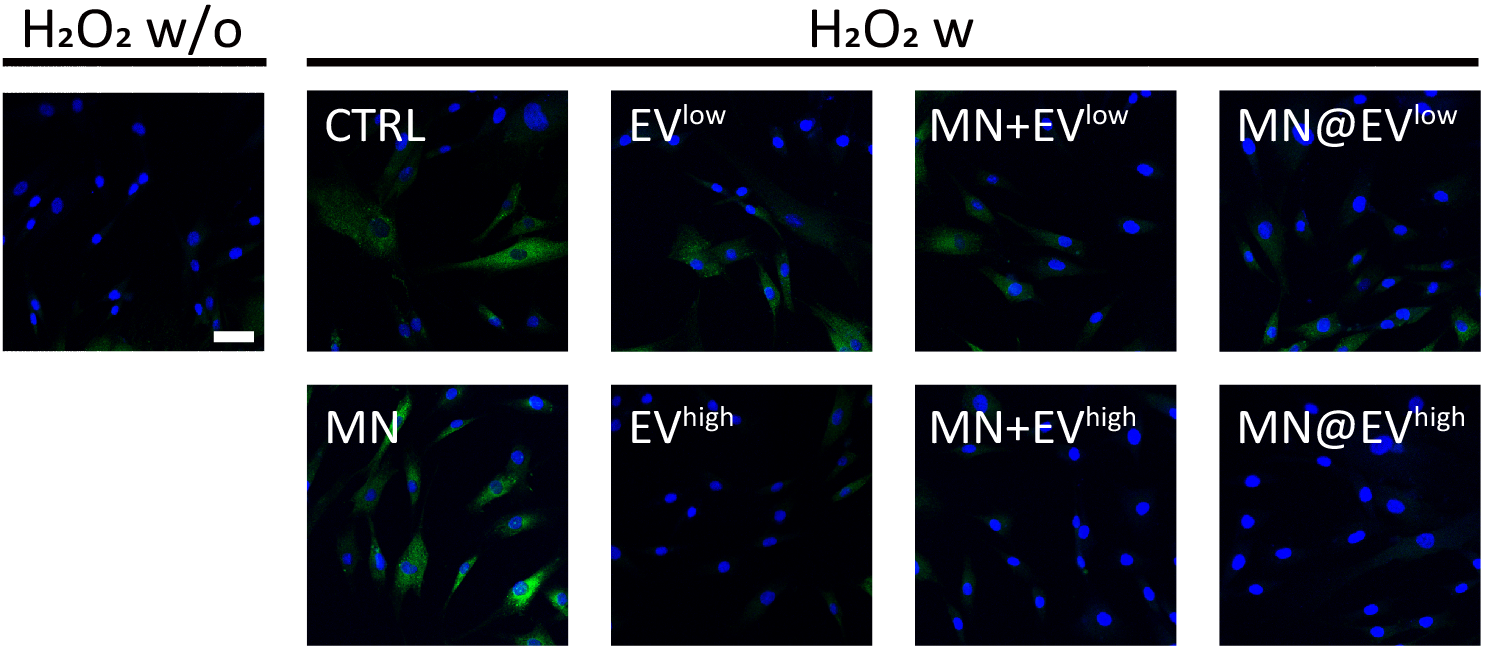


**Fig. S20** Protective effect of EV on H_2_O_2_-induced damage in DFP cells. DFPs were exposed (H_2_O_2_ w) or not exposed (H_2_O_2_ w/o) to 150 μM H_2_O_2_ for 6 h before staining with fluorescence dye. Images were captured using CLMS. Scale bar, 50 μm


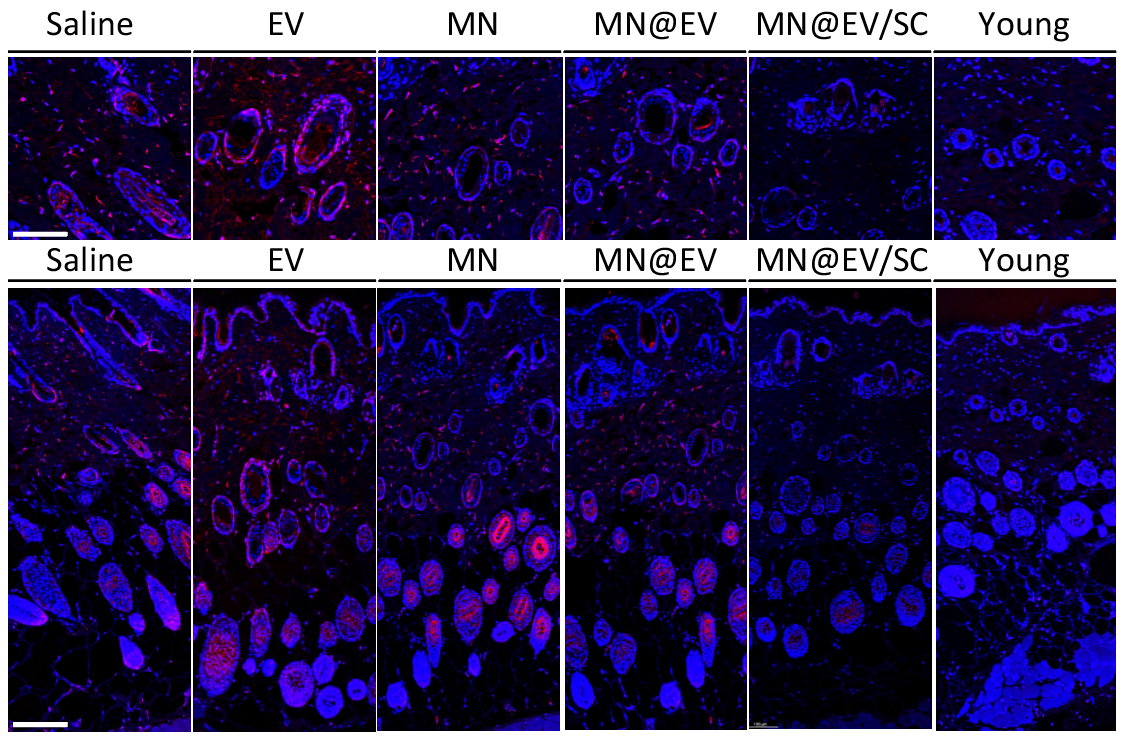


**Fig. S21** Immunofluorescence staining of p16 (red) and nuclei (DAPI, blue) in dorsal skin tissues to evaluate senescence after MN@EV/SC treatment. Scale bar, 200 μm

Supplementary References

1. T. Bauleth-Ramos, N. El-Sayed, F. Fontana, M. Lobita, M.-A. Shahbazi et al., Recent approaches for enhancing the performance of dissolving microneedles in drug delivery applications. Mater. Today 63, 239–287 (2023). <https://doi.org/10.1016/j.mattod.2022.12.007>
2. P. Snetkov, K. Zakharova, S. Morozkina, R. Olekhnovich, M. Uspenskaya, Hyaluronic acid: the influence of molecular weight on structural, physical, physico-chemical, and degradable properties of biopolymer. Polymers 12(8), 1800 (2020). <https://doi.org/10.3390/polym12081800>
3. J. Lee, G.W. Hwang, B.S. Lee, N.J. Park, S.N. Kim et al., Artificial *Octopus*-limb-like adhesive patches for cupping-driven transdermal delivery with nanoscale control of stratum corneum. ACS Nano (2024). <https://doi.org/10.1021/acsnano.3c09304>
